# Genetic Determinants in Salmonella enterica Serotype Typhimurium Required for Overcoming *In Vitro* Stressors in the Mimicking Host Environment

**DOI:** 10.1128/Spectrum.00155-21

**Published:** 2021-12-08

**Authors:** Rabindra K. Mandal, Tieshan Jiang, Young Min Kwon

**Affiliations:** a Center of Excellence for Poultry Science, University of Arkansas System Division of Agriculture, Fayetteville, Arkansas, USA; b Cell and Molecular Biology Program, University of Arkansas, Fayetteville, Arkansas, USA; Lerner Research Institute

**Keywords:** *Salmonella*, Tn-seq, conditionally essential genes, *in vivo* fitness, host stressors, virulence genes

## Abstract

Salmonella enterica serotype Typhimurium, a nontyphoidal Salmonella (NTS), results in a range of enteric diseases, representing a major disease burden worldwide. There is still a significant portion of Salmonella genes whose mechanistic basis to overcome host innate defense mechanisms largely remains unknown. Here, we have applied transposon insertion sequencing (Tn-seq) method to unveil the genetic factors required for the growth or survival of *S.* Typhimurium under various host stressors simulated *in vitro*. A highly saturating Tn5 library of *S.* Typhimurium 14028s was subjected to selection during growth in the presence of short-chain fatty acid (100 mM propionate), osmotic stress (3% NaCl), or oxidative stress (1 mM H_2_O_2_) or survival in extreme acidic pH (30 min in pH 3) or starvation (12 days in 1× phosphate-buffered saline [PBS]). We have identified a total of 339 conditionally essential genes (CEGs) required to overcome at least one of these conditions mimicking host insults. Interestingly, all eight genes encoding F_o_F_1_-ATP synthase subunit proteins were required for fitness in all five stresses. Intriguingly, a total of 88 genes in Salmonella pathogenicity islands (SPI), including SPI-1, SPI-2, SPI-3, SPI-5, SPI-6, and SPI-11, are also required for fitness under the *in vitro* conditions. Additionally, by comparative analysis of the genes identified in this study and the genes previously shown to be required for *in vivo* fitness, we identified novel genes (*marBCT*, *envF*, *barA*, *hscA*, *rfaQ*, *rfbI*, and the genes encoding putative proteins STM14_1138, STM14_3334, STM14_4825, and STM_5184) that have compelling potential for the development of vaccines and antibacterial drugs to curb Salmonella infection.

**IMPORTANCE** Salmonella enterica serotype Typhimurium is a major human bacterial pathogen that enters the food chain through meat animals asymptomatically carrying this pathogen. Despite the rich genome sequence data, a significant portion of Salmonella genes remain to be characterized for their potential contributions to virulence. In this study, we used transposon insertion sequencing (Tn-seq) to elucidate the genetic factors required for growth or survival under various host stressors, including short-chain fatty acids, osmotic stress, oxidative stress, extreme acid, and starvation. Among the total of 339 conditionally essential genes (CEGs) that are required under at least one of these five stress conditions were 221 previously known virulence genes required for *in vivo* fitness during infection in at least one of four animal species, including mice, chickens, pigs, and cattle. This comprehensive map of virulence phenotype-genotype in *S.* Typhimurium provides a roadmap for further interrogation of the biological functions encoded by the genome of this important human pathogen to survive in hostile host environments.

## INTRODUCTION

Nontyphoidal Salmonella (NTS), a Gram-negative bacterial pathogen, causes 93 million enteric infections, 155,000 diarrheal deaths, and 3.4 million bloodstream infections worldwide annually (1, 2). Salmonella enterica serotype Typhimurium (*S.* Typhimurium) is one of the leading causes of NTS (3, 4). Despite that Salmonella infection is an enormous global burden on disease worldwide and the first complete genome sequence of *S.* Typhimurium LT2 became available nearly 2 decades (2002) ago followed by additional complete genomes of >348,000 Salmonella strains (www.ncbi.nlm.nih.gov/pathogens), the mechanistic basis for *S.* Typhimurium genes required for *in vivo* survival is still unknown for a large portion of the genes (5, 6). Researchers have tried to delve into the pathogenesis of *S.* Typhimurium using different variations of high-throughput screening of transposon mutants based on negative selection (7–9). Chan et al. discovered 157 and 264 genes required by *S.* Typhimurium strain SL1344 for acute infection in mice (A-Mice) and survival inside macrophages (MΦ), respectively, using a microarray-based tracking method (9). Lawley et al. used the same method to identify 118 genes of *S.* Typhimurium SL1344 required for long-term persistent infection in mice (P-Mice) using spleen samples collected 28 days postinfection (8). Additionally, Chaudhuri et al. comprehensively assigned a core set of 611 genes of *S.* Typhimurium strain ST4/74 required for effective gut colonization in calves, pigs, and chickens (10). Recently, Silva-Valenzuela et al. identified 224 mutants of *S.* Typhimurium 14028s that were negatively selected using two pools of single-gene deletion mutants recovered from spleen and liver at 2 days postinfection in mice (Sp-Liv) (11). Previously, our laboratory conducted transposon insertion sequencing (Tn-seq) screening to identify an overlapping set of 105 coding genes of *S.* Typhimurium 14028s required for *in vitro* growth in diluted Luria-Bertani (dLB) medium, LB medium plus bile acid, and LB medium at 42°C (12). However, there is still a gap in the above approaches to correlate the genes required for growth or survival by *S.* Typhimurium between *in vitro* and *in vivo* conditions, which will help us delve into the biochemical and/or molecular basis of virulence and potentially pave a roadmap toward the development of novel vaccines, antibiotics, and/or control strategies.

In this study, we conducted Tn-seq analysis of *S.* Typhimurium 14028s under the five *in vitro* conditions mimicking host stressors found during enteric and systemic infection. Tn-seq is a powerful tool for functional analysis of bacterial genomes based on the use of random transposon mutagenesis and next-generation sequencing technology (7, 13, 14). We have applied a highly efficient method for Tn-seq library preparation that requires only a small amount of DNA without the need for enzymatic digestion or physical shearing of genomic DNA (15–18). To cause enteric infection, *S.* Typhimurium has to overcome host insults, such as low acidic pH in the stomach, osmotic pressure, and short-chain fatty acid (SCFAs) in the intestinal tract (19–22). Eventually, for systemic infection, *S.* Typhimurium has to vanquish macrophage stresses, such as oxidative stress, starvation, and hyperosmotic conditions (23–25). We hypothesized that a comparative analysis of the comprehensive sets of the *in vitro* fitness genes (for resistance against host stressors [from this and previous studies]) and *in vivo* fitness genes (required for enteric and systemic infection in different hosts [from previous studies]) will allow a better understanding of the mechanistic basis of the genetic determinants of *S.* Typhimurium required for host infection and provide enhanced resolution to link genotype to phenotype. Thus, we performed Tn-seq screenings under the five different host stressors simulated *in vitro*, which was then followed by a comparative analysis between the *in vivo* and *in vitro* fitness genes identified from previous studies and the current study.

## RESULTS AND DISCUSSION

### Stress conditions used for the Tn-seq screenings.

Among various stressors in the host environment, we selected five conditions for Tn-seq screenings in this study. They include NaCl, propionate (PA), H_2_O_2_, pH 3, and starvation, representing high osmolarity (intestinal tract), SCFAs (intestinal tract and macrophages), oxidative stress (macrophages), extreme acid (stomach and macrophages), and limited nutrients (macrophages), respectively, which *S.* Typhimurium encounters in different tissues during the course of infection in the host (26). For NaCl, propionate, and H_2_O_2_, we used growth-based selection, as these stressors at the range of concentration found in the host tissues are bacteriostatic. On the contrary, pH 3 and starvation operate as the bactericidal stressors *S*. Typhimurium encounters under conditions where the pathogen does not multiply, such as acidic stomach and acidified vacuole in macrophages (26). Therefore, survival-based selection was used for pH 3 and starvation stressors. To determine the concentrations for the growth-based selections, we performed growth assays in LB medium containing each stressor at the concentrations commonly used to mimic host stressors in the literature. The growth curves of *S.* Typhimurium 14028s wild-type strain in the presence of each of the three stressors at the respective concentrations used in the Tn-seq screenings in this study are shown in Fig. S2 in the supplemental material.

### Overall evaluation of resulting Tn-seq profiles.

**FIG 1:**
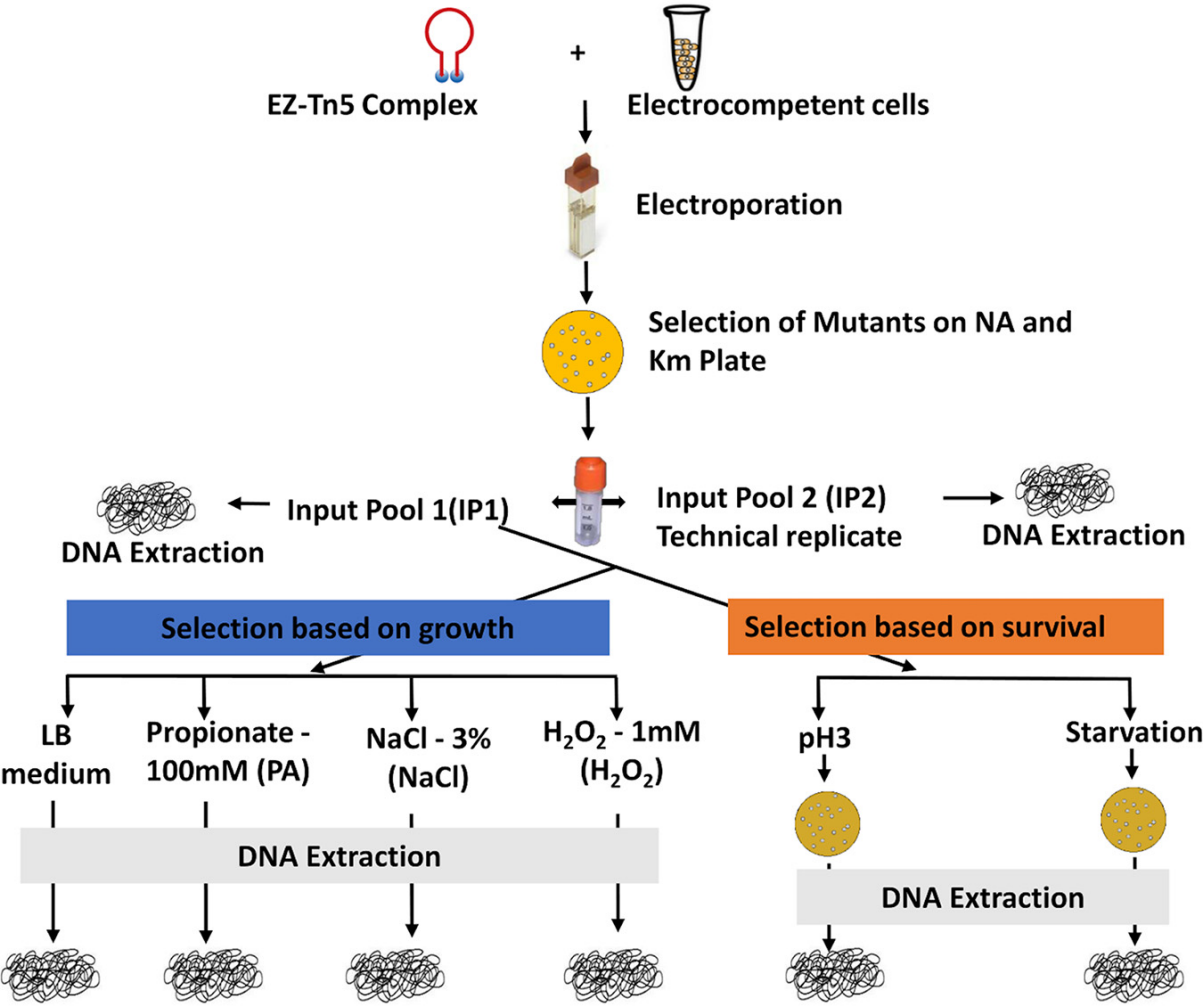
Schematic overview of the experimental design. A highly saturating Tn5 library was constructed through electroporation of EZ-Tn5 transposome complex to *S.* Typhimurium 14028s. Approximately 350,000 Tn5 mutants were collected on LB (Km + NA) plates. Complex Tn5 mutant library (IP1) was selected based on growth (LB medium [LB], 100 mM propionate in LB medium [PA], 3% NaCl in LB medium [NaCl], and 1 mM hydrogen peroxide in LB medium [H_2_O_2_]) and survival (exposed to pH 3 for 30 min [pH 3] and incubated for 12 days in 1× PBS [starvation]). Input pool 2 (IP2) was a technical replicate of input pool 1 (IP1).

We have constructed a highly saturated transposon mutant library of *S.* Typhimurium 14028s with approximately 350,000 transposon mutants created via transformation of the EZ-Tn5 transposome complex to electrocompetent cells. The complex Tn5 library, Input pool 1 (IP1), was then subjected to negative selection under the *in vitro* stress conditions encountered by *S.* Typhimurium during enteric and systemic infection as described in Materials and Methods. Input pool 2 (IP2) was the technical replicate of IP1 to evaluate the reproducibility of our Tn-seq method (Fig. 1). The Tn-seq amplicon library for Illumina sequencing was prepared for each of the input and output pools (Fig. S1A and S1B). This efficient Tn-seq protocol was developed in our laboratory and offers distinct advantages over other Tn-seq library preparation methods, including a small amount (∼100 ng) of DNA required and no need for physical shearing or restriction digestion (7, 15–17).

Illumina sequencing using a HiSeq 3000 produced 163,943,475 reads from a single flow cell lane. The raw reads were demultiplexed, allowing a perfect match for the sample barcodes used (Table S1) with the exception of up to two mismatches within the Tn5 mosaic end (ME) using a custom Perl script. H_2_O_2_ (19,250,956) had the highest number of reads followed by IP1 (10,842,764), starvation (9,518,226), IP2 (6,345,173), LB (5,004,934), pH 3 (3,841,401), PA (2,113,033), and NaCl (1,970,072) (Fig. S3A).

After demultiplexing, Illumina reads were trimmed of barcode and transposon sequences. The Tn5 junction sequences of 20 bp were extracted and mapped to the complete genome of *S.* Typhimurium 14028s (NC_016856.1) using Bowtie. The overall alignment rate throughout all Tn5 libraries was 85.19% (standard error [SE] ± 1.79). Additionally, we looked for unique insertion sites in the genome in each library. IP1 had the highest number of unique insertions (186,621) followed by LB (157,915), H_2_O_2_ (149,752), IP2 (149,740), PA (127,722), NaCl (125,918), starvation (118,607), and pH 3 (92,008) (Fig. S3A). Similarly, H_2_O_2_ had the highest average read per unique insertion site in the genome (96.007 ± 1.11) with 40 median reads, whereas NaCl had the lowest (13.53 ± 0.99) with 5 median reads (Fig. S3A).

Prealigned reads of the Tn5 library in default SAM mapping file format were fed to the “analysis of high-resolution transposon-insertion sequences technique” (ARTIST) pipeline (27). Tn5 insertions were mapped into 100-bp genome-wide windows. We observed the highest Spearman correlation coefficients (a commonly used numerical measure to describe a statistical relationship between two variables) between IP1 and IP2 and IP1 and LB (0.98, *P* < 0.0001). However, there was a lower Spearman correlation of IP1 with NaCl (0.97, *P* < 0.0001), PA (0.96, *P* < 0.0001), and H_2_O_2_ (0.93, *P* < 0.0001). We observed the lowest correlation of IP1 with pH 3 and starvation (0.84 and 0.91, respectively, *P* < 0.0001) (Fig. S3B). These relationships corroborate well with the Tn5 library selection strategies used, with a higher correlation for the selections based on growth fitness (NaCl, PA, and H_2_O_2_) and a lower correlation for the selections based on survival (pH 3 and starvation).

Additionally, we looked for the occurrence of any hot spots of Tn5 insertions in the sample libraries. We found an even distribution of Tn5 insertion reads across the libraries throughout the genome. Some of the genomic regions lacking insertions have white stripes that are clearly visible (Fig. S4) across all the samples that represent essential loci in the *S.* Typhimurium 14028s genome.

### Identification of conditionally essential genes.

In this study, we used two strategies to identify conditionally essential genes (CEGs) of *S.* Typhimurium to overcome host stressors. The first strategy was a negative selection of the complex Tn5 mutant library based on growth fitness under mild stressors (3% NaCl, 100 mM propionate, 1 mM H_2_O_2_), and the second strategy was based on survival under harsher stressors (12 days of starvation and pH 3), as shown in Fig. 1.

The ARTIST pipeline can identify if genes are entirely essential or domain essential under a given condition. In our study, only a few of the genes were identified as domain essential, and the majority of them were entirely essential. For simplicity, we assigned both categories of the genes entirely essential and domain essential into one category, CEGs. We deliberately compared each of the output pool PA, NaCl, and H_2_O_2_ with both IP1 and LB separately. As expected, most of the CEGs were overlapped with these two comparisons. For the conditions PA, NaCl, and H_2_O_2_, we considered the common set of identified CEGs via the comparison of output libraries with both IP1 and LB as CEGs for each condition. However, the output libraries for pH 3 and starvation were compared only with IP1 because the selection of the Tn5 library was based on survived mutants, and the mutant cells did not multiply during selection in liquid medium.

**FIG 2:**
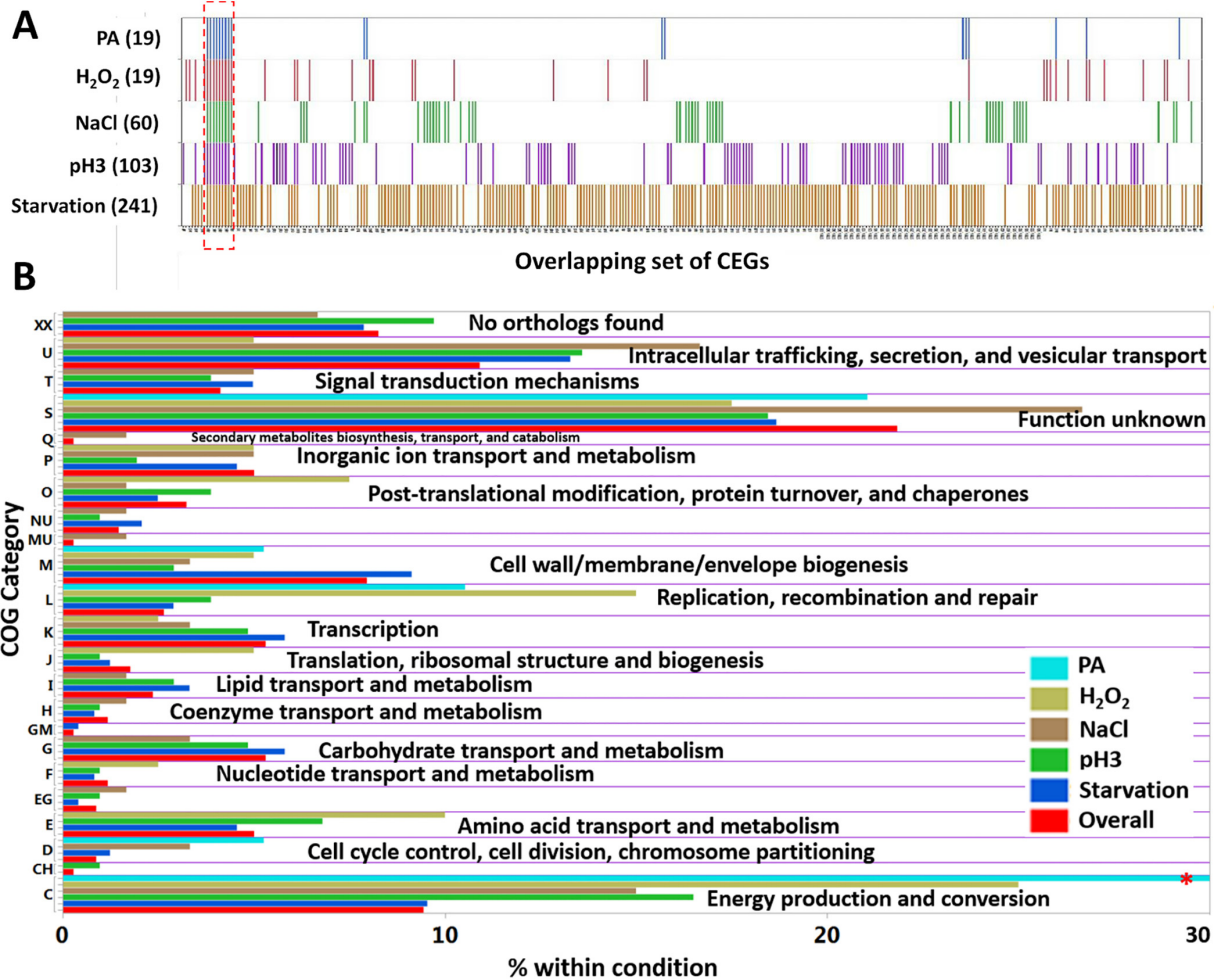
Conditionally essential genes (CEGs) of *S.* Typhimurium 14028s and cluster of orthologous groups (COG). (A) Distribution of the 339 CEGs required for fitness under at least one of the five stress conditions. Numbers inside the bracket indicate the number of CEGs identified. The red dashed box indicates the CEGs (ATP synthase genes) common to all five conditions. (B) Functional assignments of CEGs into COG category. Overall is the COG assigned to all the 339 CEGs. The red asterisk indicates that the abundance of COG C in PA was 57.89%.

We identified a total of 339 CEGs that are required for the fitness of *S.* Typhimurium 14028s under at least one of the five conditions (Fig. 2A). Starvation identified the highest number of CEGs (241), followed by pH 3 (103), NaCl (60), H_2_O_2_ (40), and PA (19), as shown in Tables S2 and S3. This might likely reflect that starvation is a severe stressor involving diverse genetic pathways for survival, while PA is a mild stressor for the fitness of *S.* Typhimurium. More than one-half of CEGs were on the lagging strand (56.63%), which is somewhat contrary to the responsive genes in Escherichia coli and Streptococcus pneumoniae (28, 29). We assigned a functional role to 96 CEGs that were putative proteins and 21 CEGs belonging to hypothetical proteins. The stress-tolerant proteins commonly identified in at least two of the *in vitro* stressors included ATP synthase, a transcriptional regulator, 3-dehydoroquinate synthase, site-specific tyrosine recombinase *xerC*, flavin mononucleotide phosphatase, ribulose-phosphate 3-epimerase, and DNA-dependent helicase II among others (Tables S2 and S3).

Intriguingly, we found that many genes in the Salmonella pathogenicity islands (SPI) were required for fitness in the presence of the *in vitro* stressors used in this study. Numerous genes in SPI-1, SPI-2, SPI-3, SPI-5, SPI-6, and SPI-11 were required for resistance against starvation (*n* = 68), NaCl (*n* = 28), and pH 3 (*n* = 27) (Table S4). However, no SPI genes were required for fitness in PA and H_2_O_2_. SPI-5 and SPI-11 genes were only conditionally essential in pH 3 (*n* = 4 and 6, respectively), while SPI-3 genes were only conditionally essential in NaCl (*n* = 7) and SPI-6 genes in starvation (*n* = 7). Tn-seq profiles for the SPI-1 region are shown in Fig. S5A as an example.

For broader insight into pathways involved in stress resistance, we assigned each CEG to the cluster of orthologous groups (COG) using the evolutionary genealogy of genes: nonsupervised orthologous groups (eggNOG) database (30). The CEGs having a top hit for the COG in *S.* Typhimurium LT2 were kept, and CEGs with no orthologous group were allotted to group XX (Fig. 2B; Table S3). Overall, 21.83% of CEGs belonged to the category “function unknown”, followed by “intracellular trafficking, secretion, and vesicular transport” (10.91%), “energy production and conversion” (9.44%), and “no orthologs found” (8.26%) among others. A substantial portion of CEGs (30.6%) falling into either “function unknown” or “no orthologs found” shows that our data set is rich in novel genotype-phenotype relationships.

Additionally, we were interested to see if any CEGs identified in our study fell into the essential genomes of *S.* Typhimurium in other strain backgrounds. Essential genomes of *S.* Typhimurium strain SL3261 (selected on LB agar) (31) and *S.* Typhimurium strain LT2 (selected on rich medium) (32, 33) were compared with the CEGs of *S.* Typhimurium 14028s identified in this study. Genes in different strain backgrounds were examined for the corresponding orthologous genes in the *S.* Typhimurium 14028s background. Interestingly, 10 and 15 CEGs in this study were shared with the essential genes of *S.* Typhimurium SL3261 and LT2, respectively (Table S5 and Fig. S6). This indicates that these genes that are essential in other strain backgrounds are dispensable in the *S.* Typhimurium 14028s strain background.

### Phenotypic basis for the requirement of CEGs in *S.* Typhimurium.

Next, we delved into the phenotypic mechanisms related to the CEGs identified in our study. For convenience, we split the section into specific CEGs required for fitness in only one stressor and common CEGs shared in at least two stressors out of five host stressors.

**FIG 3:**
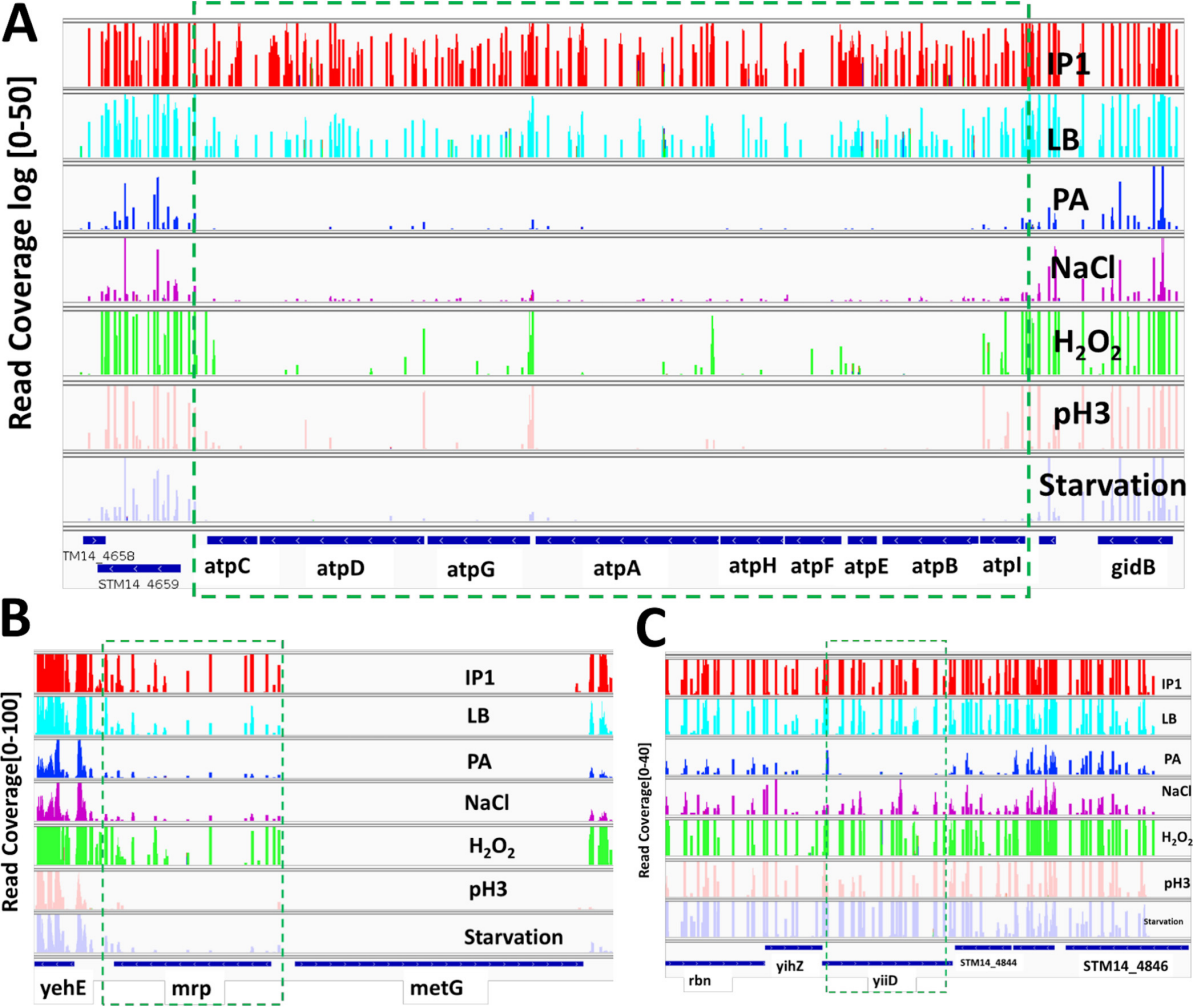
Tn-seq profiles for selected genes across 7 conditions. Along the *y* axis, the numbers in the bracket indicate the raw read coverage. (A) ATP synthase genes conditionally essential under all five conditions (PA, NaCl, H_2_O_2_, pH 3, and starvation). (B) Gene *mrp* essential in pH 3 and starvation. (C) Gene *yiiD* essential in PA only.

**(i) CEGs specifically required for propionate (100 mM PA) stress resistance.** CEGs specific for the fitness of *S.* Typhimurium in propionate were *yiiD* and *sdhAD* (Fig. S7). YiiD is a putative acetyltransferase protein (read coverage shown in Fig. 3C). Acetylation, a posttranslation protein modification, was previously shown to enable prokaryotes to increase stress resistance (34). Additionally, succinate dehydrogenase flavoprotein (*sdhA*) and cytochrome *b*_566_ (*sdhD*) subunit proteins were upregulated by intestinal SCFA in *S.* Typhimurium (35). Chowdhury and Shimizu reported that *sdhA* in the tricarboxylic acid cycle (TCA) was highly induced during temperature upshift in E. coli (36).

**(ii) CEGs specifically required for osmotic (3% NaCl) stress resistance.** Twenty-six resistance genes of *S.* Typhimurium were required for fitness in osmotic stress (3% NaCl) alone (Fig. S7). Protein-protein network analysis using STRING database version 11.5 (http://string-db.org) against S. enterica LT2 showed three distinct clusterings of genes, SPI-3 (*mgtBC*, *misL*, *cigR*, *slsA*, *fidL*, and *marT*), two-component system (TC; *dcuBRS*), and sodium ion transport (*yihPO*) along with other nodes (https://version-11-5.string-db.org/cgi/network?networkId=bLmjZQ9kZpsE). SPI-3 genes are important for intracellular replication inside the phagosome, where Salmonella experiences hyperosmotic stress (37). The virulence genes *mgtC* and *mgtB*, Mg^2+^ were expressed 5-fold higher when *S.* Typhimurium was exposed to 0.3 M NaCl (38). MisL, an autotransporter protein is an intestinal colonization factor (activated by *marT*, a transcriptional regulator) that binds to extracellular matrix fibronectin in an animal host and is also involved in adhesion to plant tissue (39). Deletion of *cigR* in Salmonella enterica serotype Pullorum resulted in significantly decreased biofilm formation and increased virulence (40). Additionally, Figureueira et al. showed that the Δ*cigR* strain of *S.* Typhimurium had attenuated replication in mouse bone marrow-derived macrophages (41).

*yihPO* genes are essential for capsule assembly that is required by Salmonella for environmental stress persistence, such as desiccation (42). The absence of *ompL* (ortholog of *yshA*) leads to solvent hypersensitivity, as it helps in the stabilization of cell wall integrity protecting from solvent penetrance as a physical barrier (43). In E. coli, genes under the control of *dcuS*-*dcuR*, a two-component system, were not affected following hyperosmotic shock (44). However, *dcuBRS* was conditionally essential in *S.* Typhimurium for fitness during osmotic stress. Putative cytoplasmic protein (STM14_4542, STM14_4828, and STM14_5175), putative inner membrane protein (STM14_4824 and STM14_5184), and putative hydrolase (STM14_4823) were also required for osmotic stress tolerance.

**(iii) CEGs specifically required for oxidative (1 mM H_2_O_2_) stress resistance.** We identified 16 specific resistance genes required for the fitness of *S.* Typhimurium in the presence of 1 mM H_2_O_2_ (Fig. S7), and the functional protein association network analysis among the genes was constructed using STRING against S. enterica LT2 (https://version-11-5.string-db.org/cgi/network?networkId=b5BBHR89UGpJ). Major resistance genes were those involved in the two-component system (*glnD*, *rpoN*, *arcA* [STM4598], and *arcB* [STM3328]), DNA recombination (*recJ* and *xerD*), and metal ion transport (*corA* and *trkA*).

Hydrogen peroxide kills E. coli cells with two distinct modes: mode 1 killing occurs at a lower concentration of H_2_O_2_ due to DNA damage, and mode 2 killing occurs at a higher concentration of H_2_O_2_ due to damage of other structures such as proteins and lipids (45). Nucleic acid metabolic process genes involved in oxidative stress resistance were *recJ*, *xerD*, *sun*, and *rpoN*. *RecJ* protein, a single-stranded DNA (ssDNA)-specific 5′ to 3′ exonuclease/deoxribophophodiesterase, plays a role in homologous recombination, mismatch repair, and base excision repair (46). In E. coli, *xerD*-knockout mutants are hypersensitive to tightly bound DNA-protein complexes (TBCs) that block replication forks *in vivo* (47). *rpoN*, the alternative sigma factor 54 (σ^54^), is an important regulator of stress resistance and virulence genes in many bacterial species (48). σ^54^ is involved in carbon/nitrogen limitation, nucleic acid damage, the cell envelope, and nitric oxide stress (49). However, Hwang et al. found that an *rpoN* mutant in Campylobacter jejuni was more resistant to 1 mM H_2_O_2_ (50).

Additionally, cellular component genes crucial for fitness in H_2_O_2_ stress were *dsbC*, *glmS*, *trkA*, and *corA*, including *sun* and *xerD*. *DsbC*, a protein essential for disulfide bond isomerization in the periplasm, has a new role in E. coli in protection against oxidative stress (51). In E. coli, *GlmS* plays an important role in cell wall synthesis, thus protecting cell envelope stress response (52). HscB, a chaperone-encoding gene is upregulated after exposure to oxidative stress in Burkholderia pseudomallei (53). YbgF, an outer membrane vesicle protein, increases the survival of bacteria during exposure to stress or from toxic unfolded proteins by releasing the unwanted periplasmic component (54).

**(iv) CEGs specifically required for higher acidic (pH 3) stress resistance.** We found 49 specific stress resistance genes required only for the survival of *S.* Typhimurium under extreme acidic conditions (pH 3) among other stressors (Fig. S7). Formate dehydrogenase (*fdoHI* and *fdhDE*), curli proteins (*csgBDEFG*), virulence and envelope proteins (SPI-2 [*orf245*, *orf408*, *ssaB*], SPI-5 [*pipBC*, *sopB*], and SPI-11 [*envEF*, *pagCD*, *msgA*, and STM14_1486 where *ssaB*, *pipB*, and *sopB* are effector proteins]), and biopolymer transport protein (*exbD* and *exbB*) were clustered in functional protein association network analysis using STRING (https://version-11-5.string-db.org/cgi/network?networkId=bS96Nh4Mgvxr).

Formate dehydrogenase catalyzes the oxidation of formate (HCOO^−^) to CO_2_ and H^+^. The released electrons from this reaction are used by two cytoplasmic protons to form dihydrogen, thus consuming net protons, consequently, counteracting acidification (55). Curli is a major complex extracellular proteinaceous matrix produced by *Enterobacteriaceae* that helps pathogenic bacteria like Salmonella in adhesion to surfaces, cell aggregation, and biofilm formation (56). Acidic pH strongly enhances biofilm formation in Streptococcus agalactiae (57). We hypothesize that curli fibers might potentially protect bacteria from severe acid stress through the physical barrier and likely by the generation of alkaline compounds, as in oral biofilms (58). PhoP regulates SPI-11 genes, such as *envEF*, *pagCD*, and *msgA*, where the latter three are required by Salmonella to survive low pH within macrophages (59, 60). In Helicobacter pylori, the only organism to colonize in the acidic human stomach, the *ExbB*/*ExbD*/*TonB* complex is required for acid survival and periplasmic buffering (61). Additionally, survival of Δ*exbD* was diminished compared to the wild type at pH 3 in E. coli (62). The *metC* gene encoding a key enzyme in methionine biosynthesis, required for the generation of homocysteine, pyruvate, and ammonia, plays a crucial role in bacterial acid stress responses (63). However, there was no overlap between Salmonella enterica serotype Derby genes identified by Gu et al. for growth under acidic conditions due to different experiment design (growth versus survival) and serotypes (64).

**(v) CEGs specifically required for starvation stress resistance.** Out of 241 Salmonella fitness genes essential for starvation stress, 160 genes were important for resistance against only starvation stress among the five infection-relevant conditions in this study (Fig. S7) (https://version-11-5.string-db.org/cgi/network?networkId=b1V1bvEGNU4v). Major enriched gene pathways were oxidative phosphorylation, pathogenesis, two-component system, and lipopolysaccharide biosynthetic process among others. NADH dehydrogenase, the first component of the respiratory chain, subunit proteins (*nuoCEFGHLMN*) were required for the fitness of Salmonella during long-term carbon starvation. Salmonella defective in NADH dehydrogenase enzyme exhibits defective energy-dependent proteolysis during carbon starvation (65). Proteolysis of unbound or unemployed proteins helps bacteria to access nutrients as an important survival strategy during carbon starvation (66). SPI-1 (*hilACD*, *iagB*, *invH*, *orgAC*, *prgHIJK*, STM14_3500, and STM14_3501) and SPI-2 (*ssaMNOPQRSTV*, *sscB*, and *sseDEF*) encoding type III secretion system (T3SS) and SPI-6 (*safABCD*, *sinR*, STM14_0359, and *ybeJ*) encoding type VI (T6SS) secretion system were required for *in vitro* survival in long-term starvation stress. Salmonella usually requires SPI-1 genes for the invasion of intestinal epithelial cells (67). HilACD regulates SPI-1 invasion gene expression under multiple environmental conditions, including stationary phase, pH, osmolality, oxygen tension, and short-chain fatty acids (68). SPI-2 genes are expressed under *in vitro* starvation conditions, indicating the use of nutritional deprivation as a signal (69). T6SS has been hypothesized to confer a growth advantage to bacteria in environmental niches where bacterial competition for a nutrient is critical for survival (70).

Two-component systems (TCs), a basic stimulus-response coupling mechanism, enable microbes to respond to various stimuli, such as pH, osmolality, quorum signals, or nutrient availability, and regulate their cellular functions (71). TCs required for fitness under starvation conditions were *envZ*/*OmpR*, *cpxA*/*cpxR*, sensory histidine kinase protein (*phoQ*), and *kdpD* (Fig. S5B). EnvZ/*OmpR* regulates the synthesis of porin proteins (*ompF* and *OmpC*) that are important for the survival of E. coli in seawater under starvation stress conditions (72). It is believed that carbon starvation causes cell envelope stress. Bacchelor et al. found that *cpxA*/*cpxR* in E. coli regulates the expression of porins *ompF* and *ompC*, a major component of the outer membrane. However, Kenyon et al. showed that starvation stress of *S.* Typhimurium does not require cpxR-regulated extracytoplasmic functions (73, 74). Two genes *phoQ* and *kdpD* play a role in Mg^2+^ and K^+^ homeostasis, respectively, which is critical to the virulence and intracellular survival of *S.* Typhimurium (71, 75).

The outer membrane of Gram-negative bacteria contains phospholipids and lipopolysaccharides (LPS). LPS molecules act as a permeability barrier to prevent the entry of toxic compounds and allow the entry of nutrient molecules (76). LPS biosynthetic process genes required for fitness under starvation conditions were *rfbABCD*, *rfbUNMKP*, *galF*, *udg*, *wzxE*, and *wzzB*. Starvation of carbon energy sources activates an envelope stress response in *S.* Typhimurium (77). Additionally, *pstSCAB*, encoding the Pst ABC transporter, catalyzes the uptake of inorganic phosphate (78). Mutations in the Pst system results in structural modifications of lipid A and an imbalance in unsaturated fatty acids, consequently leading to an increase in outer membrane permeability, making E. coli more vulnerable to environmental stresses, including antimicrobial peptides and low pH (78).

Additional genes required for starvation stress resistance were *aroGH*, ytfMNP (ytfM [outer membrane protein]), and *stcB* (putative periplasmic outer chaperone protein). Furthermore, other envelope proteins were outer membrane lipoproteins (*stcD* and *yifL*), putative outer membrane proteins (*stcC*, STM14_0404, and *ytfM*), and putative inner membrane proteins (STM14_0398, STM14_0402, STM14_2763, STM14_4741, STM14_4742, STM14_4745, STM14_4880, *ydiK*, and *yjeT*). Similarly, putative cytoplasmic proteins required for starvation stress were STM14_2759, STM14_4743, STM14_5374, *ydiL*, and *ytfP*.

**(vi) CEGs required for tolerance to multiple stressors.** We found 12 Salmonella genes required for stress resistance in either three or four of the *in vitro* host stresses in our study, as shown in a STRING protein-protein interaction network (https://version-11-5.string-db.org/cgi/network?networkId=bVnBdnBjt1SH). The enriched gene ontology (GO) biological process/KEGG pathways were noncoding RNA (ncRNA) processing (*gidAB* and *mnmE*), DNA metabolic process (*dam*, *uvrD* [SOS response], and *xerC*), and biosynthesis of amino acids (*aroB* and *rpe* [microbial metabolism in diverse environments]). Also, other responsive proteins included ATP synthase subunit protein (*atpI*), putative permease (STM14_4659), inner membrane protein (*damX*), and flavin mononucleotide phosphatase.

*damX*, *dam*, *rpe*, *aroB*, *uvrD*, and *yigB* were required for fitness under pH 3, starvation, and H_2_O_2_. Disruption of *damX* in S. enterica causes bile sensitivity (79). The DNA adenine methylation gene (*dam*) plays an important role in bacterial gene expression and virulence (80). A dam mutant of S. enterica is extremely attenuated in mice (81). The gene *aroB* encodes dehydroquinate synthase, a part of the shikimate pathway, which is essential for bacteria and is absent in mammals (82). In prokaryote species, *uvrD* is involved in maintaining genomic stability and helps DNA lesion repair, mismatch repair, nucleotide excision repair, and recombinational repair (83). Overproduction of *yigB* produced higher-level persister cells that exhibit multidrug tolerance in E. coli (84). However, deletion of *gidB* (glucose-inhibited division gene B) confers high-level antimicrobial resistance in Salmonella and has compromised overall bacterial fitness compared to wild type (85). GidA (together with *mnmE*) is responsible for the proper biosynthesis of 5-methylaminomethtyl-2-thouridine of tRNAs, and deletion causes attenuation in bacterial pathogenesis (86). In addition, *mrp* gene involved in thiamine synthesis was shown to be required for survival in both pH3 and starvation (Fig. 3B).

### ATP synthase genes are obligatory for Salmonella fitness during *in vitro* host stressors.

ATP synthase (F_o_F_1_-ATPase) is a ubiquitous enzyme largely conserved across all domains of life. All of the eight genes encoding ATP synthase subunit proteins were required for the fitness of *S.* Typhimurium under all five *in vitro* conditions of our study (Fig. 2A and 3A). F_o_F_1_-ATP synthase complex is required for ATP production from ADP and P_i_. ATP synthase also regulates pH homeostasis in bacteria (Listeria monocytogenes and *S.* Typhimurium) at the expense of ATP (87). In Streptococcal faecalis, upregulation of F_o_F_1_-ATPase promotes ATP-dependent H^+^ extrusion under acidic conditions. However, in E. coli, the expression of ATP synthase is decreased under acidic conditions (88). ATP synthase in Mycobacterium and Staphylococcus has been validated as a promising target for new antimicrobial drugs (87, 89).

### Mutant phenotypic assays for growth and survival.

**FIG 4:**
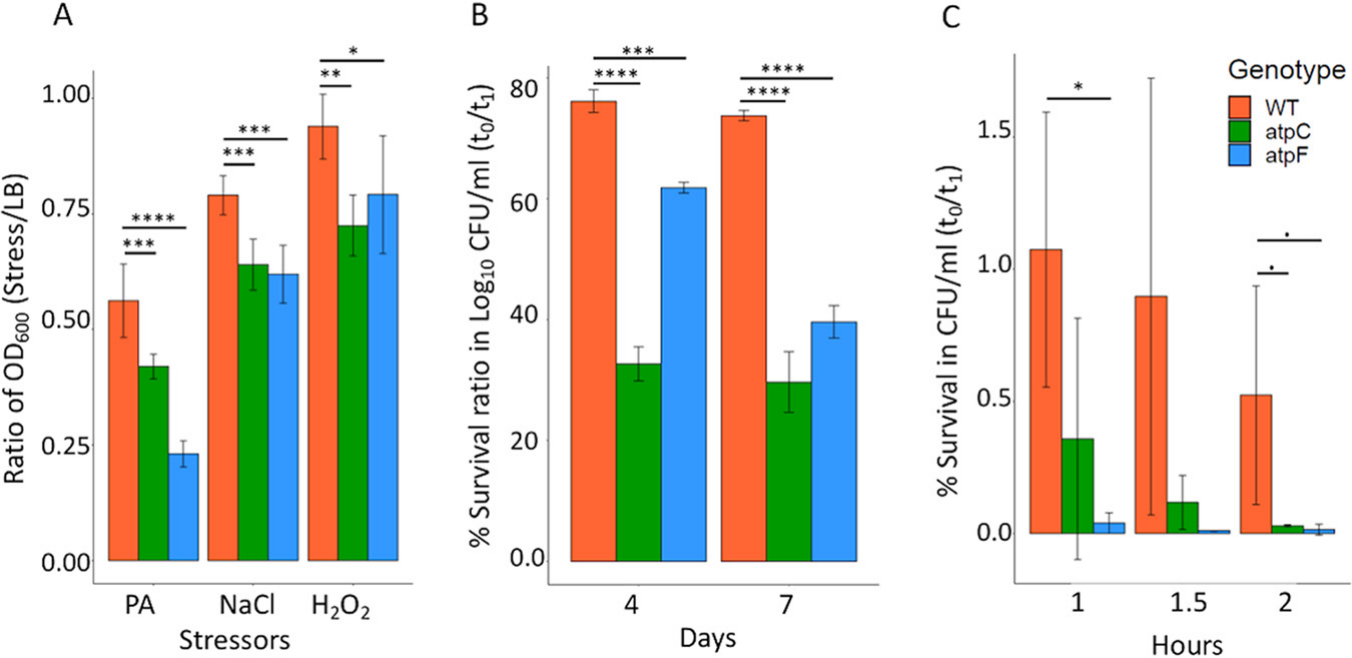
Mutant phenotypic assays for growth and survival. Growth (A) and survival assays (B and C) of wild type (WT), Δ*aptC*, and Δ*atpF*. (A) Bacteria were treated with 100 mM propionate, 3% NaCl, and 1 mM H_2_O_2_ in LB, and growth (OD_600_) was measured at 6 h. (B) Bacteria were starved for 4 (*t*_1_) days and 7 (*t*_1_) days in 1× PBS. Viable bacteria counts were enumerated by plating serial dilutions on LB agar plates. (C) Bacteria were incubated in glycine-HCl buffer (pH 3.0) and incubated for 1, 1.5, and 2 h. Viable cells were enumerated by plating serial dilutions. Bar represents mean ± SE. All of the above-described experiments were performed in ≥3 replicates. Statistical analysis was performed using one-way analysis of variance (ANOVA) with correcting for multiple comparisons using the Holm-Sidak method; *, *P* < 0.05; **, *P* < 0.01; ***, *P* < 0.001; ****, *P* < 0.0001.

In light of the importance of ATP synthase genes, two single-gene deletion mutants, Δ*aptC* and Δ*atpF*, were chosen for phenotypic validation. Both of the mutants had significantly reduced growth compared to wild-type *S.* Typhimurium in 100 M PA, 3% NaCl, and 1 mM H_2_O_2_ after 6 h of growth (Fig. 4A). In fact, growth of the two mutants was impaired in LB broth compared to the wild type. However, the growth defect was even more severe under the stressors as clearly illustrated in Fig. 3A, which allowed for identification of the eight genes in the *atp* operon as CEGs under all five stress conditions. Similarly, the survival of both mutants was significantly lower than wild-type *S.* Typhimurium after starvation in 1× phosphate-buffered saline (PBS) for 4 days and 7 days (Fig. 4B). Additionally, the survival fitness of only the Δ*atpF* mutant was significantly reduced compared to wild-type *S.* Typhimurium when incubated at pH 3 for 1 h. A trend for a decrease in survival fitness was observed for both mutants compared to the wild type when incubated for 2 h at pH 3 (Fig. 4C). For the survival assays (starvation and pH 3), sampling was performed at various time points to determine survival. However, we picked the time points that can highlight the mutant phenotypes more clearly to present the data in Fig. 4B and C. The mutant phenotypes showed the same trends in reference to the wild type to various degrees over the sampling time points.

### Phenotypic bases of Salmonella *in vivo* fitness genes required for enteric and systemic infection.

**FIG 5:**
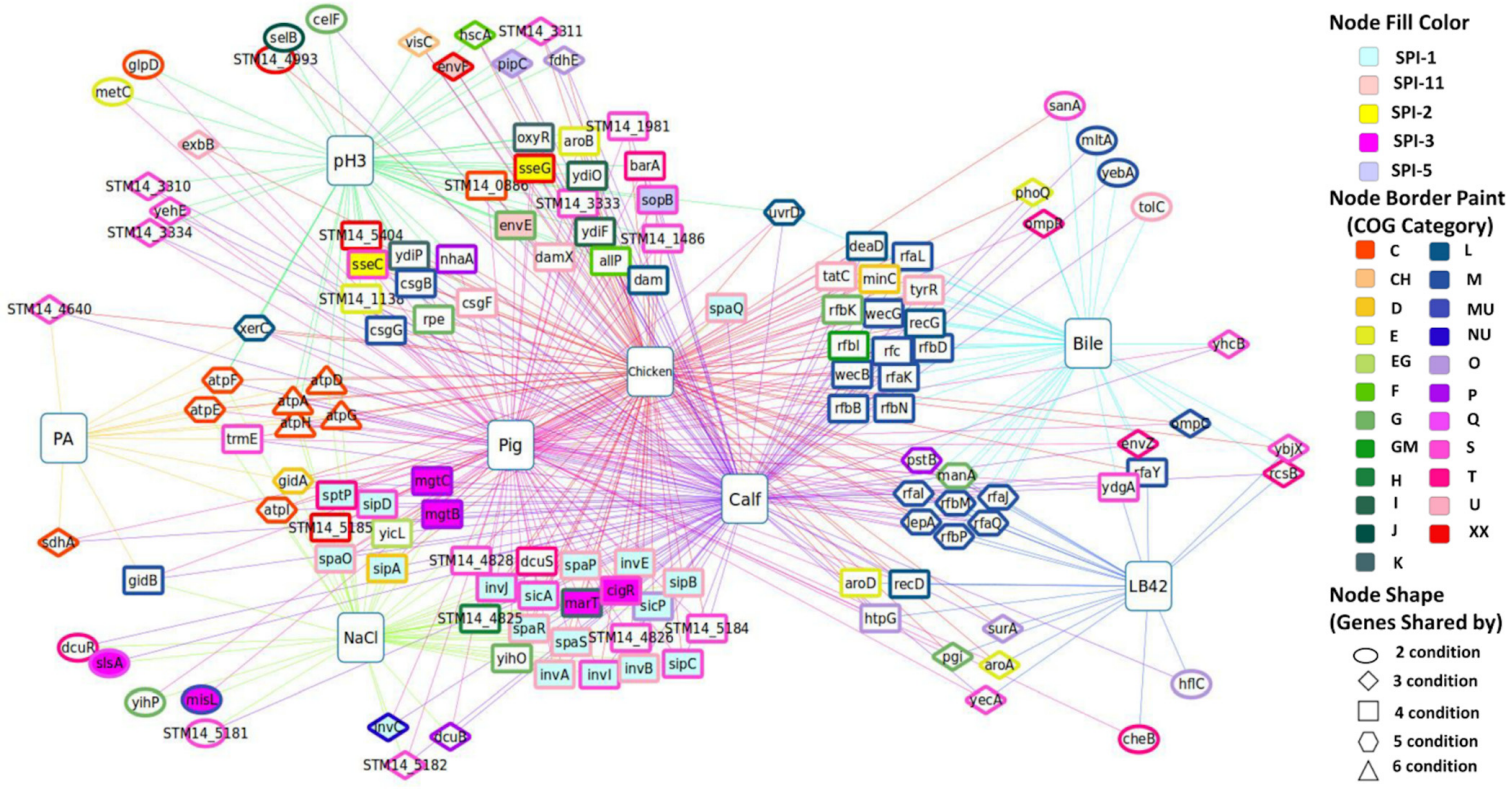
Genotype-phenotype network connections illustrating phenotypic basis of *S.* Typhimurium genetic factors required for enteric infection (*in vitro* versus *in vivo* [enteric]). Large square nodes indicate various conditions (studies), and small nodes are fitness genes. Each node (gene) is shared by at least one of the *in vitro* conditions (i.e., stressors encountered by Salmonella during enteric infection; PA, pH 3, NaCl, bile, and LB42; current study and our previous study) and at least one of the *in vivo* enteric conditions (pig, calf, and chicken; a previous study). The interactive network through the network data exchange (NDEx) is available at www.ndexbio.org/#/network/027b067d-e209-11e8-aaa6-0ac135e8bacf (118).

**FIG 6:**
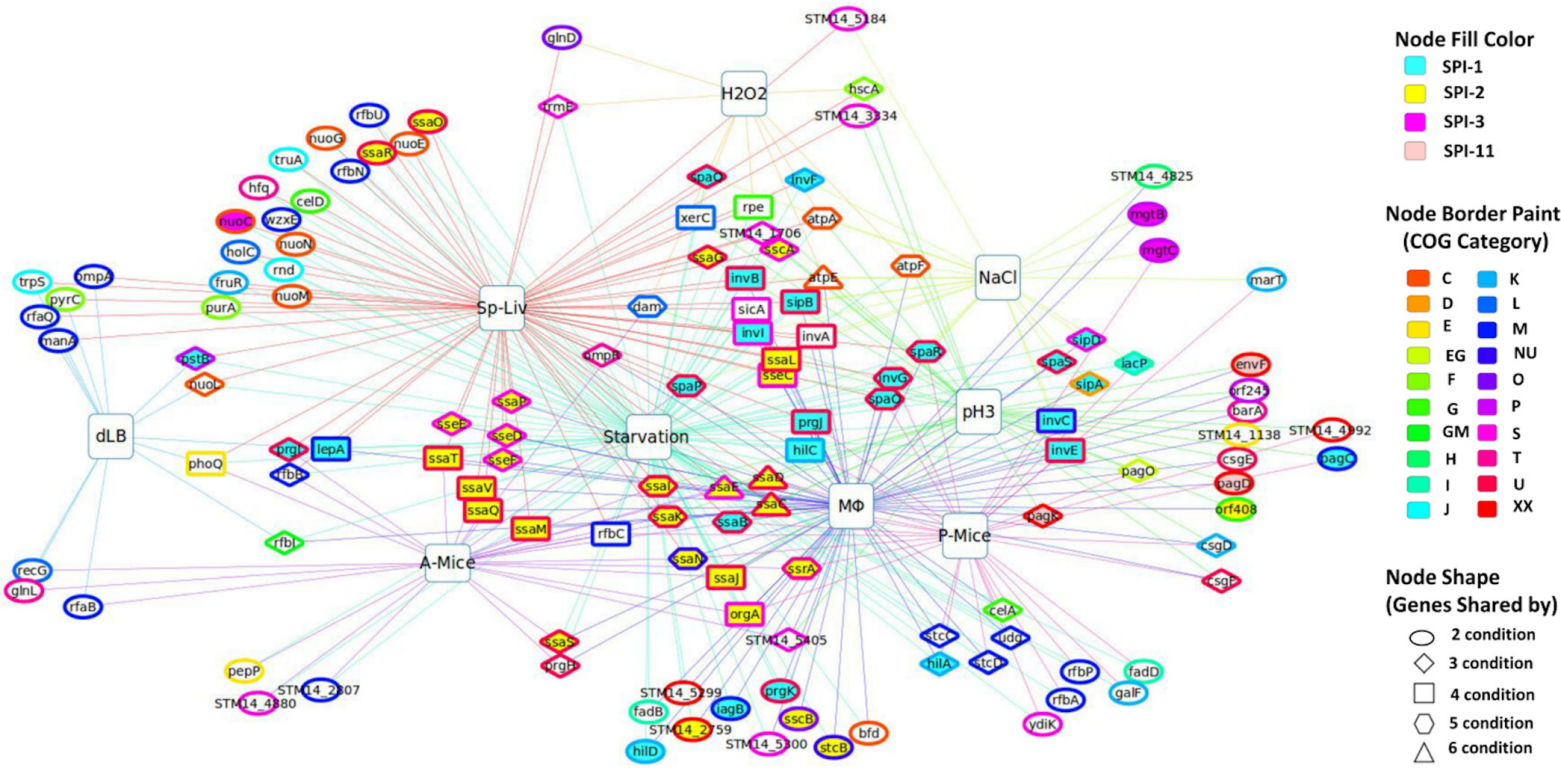
Genotype-phenotype network connections illustrating phenotypic basis of *S.* Typhimurium genetic factors required for systemic infection (*in vitro* versus *in vivo* [systemic]). Large square nodes indicate various conditions (studies), and small nodes are fitness genes. Each node (gene) is shared by at least one of the *in vitro* conditions (i.e., stressors encountered by Salmonella inside macrophages; NaCl, H_2_O_2_, pH 3, starvation, and dLB; current study and our previous study) and at least one of the *in vivo* systemic conditions (MΦ, Sp-Liv, P-Mice, and A-Mice; previous studies). The interactive network through the network data exchange (NDEx) is available at www.ndexbio.org/#/network/5e78ad70-e209-11e8-aaa6-0ac135e8bacf (118).

Numerous genes in *S.* Typhimurium that are required for *in vivo* fitness during infection in either cell culture or animal infection models have been identified in previous studies, suggesting that they are required by *S.* Typhimurium to overcome host defenses. However, for a large portion of them, the phenotypic basis by which they are required in particular *in vivo* niches remain unknown. Therefore, we constructed the genotype-phenotype network diagrams in Fig. 5 (enteric infection) and Fig. 6 (systemic infection) to show all the genes that are important for fitness under at least one of the *in vitro* conditions (this study and our previous study), which are also important for fitness in at least one of the *in vivo* infection models (previous studies from other labs). The genes that were important either under the *in vitro* or *in vivo* conditions only were excluded from the diagrams. The information on the common requirements of the genes shown in these networks (Fig. 5 and 6) for at least one well-defined *in vitro* stress and *in vivo* infection model is valuable because it provides new insights into the nature of the selective pressures *S.* Typhimurium might be facing during infection in the host and which genetic factors *S.* Typhimurium uses to overcome each particular stressor.

**(i) Enteric infection.** We have identified an overlapping set of 135 CEGs that are commonly required to cause enteric infection in at least one of the hosts (pig, calf, and chicken [10]) and for fitness in one of the *in vitro* host stressors (LB42, bile [12], pH 3, PA, and NaCl) encountered during enteric infection (Fig. 5; Table S6). Genes in SPI-1 (*invABCEIJ*, *sicAP*, *sipABCD*, *spaOPQRS*, and *sptP*) and SPI-3 (*cigR*, *marT*, *mgtBC*, *misL*, and *slsA*) were required for fitness in NaCl and all hosts. However, genes encoding SPI-2 (*sseCG*), SPI-5 (*slsA* and *pipC*), and SPI-11 (*envEF*) were essential for fitness in only one *in vitro* stressor pH 3 and intestinal colonization in three hosts. Other enriched pathways were lipopolysaccharide biosynthesis (*rfaIJKLQY* and *rfbBDKMNP*), oxidative phosphorylation (ATP synthase genes and *sdhA*), and biosynthesis of amino acids (*aroABD*, *rpe*, and *metC*) including others as shown in the STRING protein-protein interaction against S. enterica LT2 (https://version-11-5.string-db.org/cgi/network?networkId=bHPdi1Ibnskf).

High osmolality, low oxygen, and late log phase induce *hilA* expression *in vitro* that in turn regulates the expression of SPI-1 genes (90). Interestingly, we identified SPI-1 genes as fitness genes required for *in vitro* NaCl stress. Similarly, LPS biosynthetic process genes were enriched in LB42 and bile and in pig, calf, and chicken for fitness during enteric infection. LPS, a critical factor in the virulence of Gram-negative bacterial infection, is required for intestinal colonization, resistance to killing by macrophages, swarming motility, serum resistance, and bile stress (12, 91). A csgBA (curli subunit protein) mutant of *S.* Typhimurium was attenuated in its ability to elicit fluid accumulation in bovine ligated ileal loops (92) and is required for fitness at pH 3, including *csgF* and *csgG*. Additionally, putative proteins STM14_1138, STM14_1486, STM14_1981, and STM14_3333 and STM14_4826, STM14_4828, STM14_5184, and STM14_5185 (hypothetical protein) were required for fitness *in vitro* under acidic and osmotic stress, respectively, and enteric infection in the three hosts.

**(ii) Systemic infection.** We compared the CEGs that are shared between at least one of the *in vitro* host stressors (H_2_O_2_, NaCl, pH 3, starvation, and dLB [12]), encountered inside MΦ and *in vivo* systemic infections (MΦ [9], A-Mice [9], P-Mice [8], and Sp-Liv [11]) and identified an overlapping set of 130 genes (Fig. 6; Table S7) shown in a protein-protein interaction network using STRING (https://version-11-5.string-db.org/cgi/network?networkId=bqRqTdvi8p4u). SPI-1 genes (*hilACD*, *iacP*, *iagB*, *invABCEFGI*, *orgA*, *prgHIJK*, *sicA*, *sipABC*, and *spaOPQRS*) encoding T3SS were essential for fitness in NaCl, starvation, MΦ survival, and systemic infection. Additionally, SPI-2 genes (*ssaBCDEGIJKLMNOPQRSTV*, *orf245*, *orf408*, *sscAB*, *sseCDEF*, *ssrA*, and STM14_1706) encoding T3SS were required for fitness in pH 3, starvation, MΦ survival, and systemic infection. Similarly, SPI-3 genes (*marBCT*) were required for fitness in NaCl, MΦ survival, and persistent infection in mice (P-Mice). SPI-11 genes (*envF* and *pagCD*) were required for fitness in pH 3, MΦ survival, and P-Mice.

Other than SPI genes, the major enriched genes were nucleic acid metabolic process (*dam*, *trpS*, *MnmE*, *truA*, *serc*, *csgD*, *ompR*, and *cra*), lipopolysaccharide biosynthetic process (*rfbABCNPU*, *rfaB*, *udg*, and *galF*), oxidative phosphorylation (ATP synthase genes and NADH dehydrogenase genes), and two-component system (*ompR*, *barA*, *phoQ*, *glnDL*, and *pagKO*) among others (Fig. 6). The gene *dam* was required for fitness in H_2_O_2_, NaCl, A-Mice, and Sp-Liv. XerC and *rpe* were required for H_2_O_2_, pH 3, starvation, and Sp-Liv. Interestingly, *pagK* was not identified as a CEG in A-Mice, P-Mice, and Sp-Liv but in pH 3, starvation, and MΦ. Putative genes essential for either *in vitro* or *in vivo* systemic infection were STM14_1138, STM14_4880, STM14_4992, STM14_5184, STM14_2759, STM14_2807, STM14_3334, STM14_4825, STM14_5299, and STM14_5300.

## CONCLUSION

A recent study by Kroger et al. presented transcriptomes of *S.* Typhimurium under 22 distinct infection-relevant environmental conditions *in vitro*. The study found induction of Salmonella pathogenicity islands under *in vitro* conditions, such as early stationary phase, anaerobic growth, oxygen shock, nitric oxide shock, and pH 3, NaCl, bile, and peroxide shock among others (93). However, transcription of a gene does not necessarily indicate the requirement for that gene function for fitness under a given particular condition. The transcript can be a leaky expression or required for fitness in the upcoming environment through predictive adaptation, phenomena where bacteria can anticipate and preemptively respond to regular environmental fluctuations (temporally distributed stimuli) that confers a considerable fitness advantage for the survival of an organism (94, 95). Traditionally, it is believed that the “central dogma of life” that information flows from DNA to RNA to proteins is highly concordant. However, there is a modest correlation between levels of transcripts and corresponding proteins (96–98). Thus, functional genomics screening such as Tn-seq is expected to reveal more direct functional aspects of the genes involved in responding to the current stresses.

In this report, we were able to map genotype-to-phenotype links, providing the phenotypic basis of the genetic requirements for fitness for an overlapping set of 221 virulence genes for *in vivo* fitness (Fig. S8 in the supplemental material). These CEGs were required for fitness for at least one of the *in vitro* host stressors (PA, NaCl, pH 3, starvation, bile, LB42, and dLB) and enteric infection (calf, chicken, and pig) or systemic infection (mice, including intracellular survival inside macrophages). Forty-four common CEGs were required to cause both systemic and enteric infections (*in vivo* fitness) and *in vitro* fitness (Fig. S8; Table 1). Common SPI genes for *in vivo* and *in vitro* fitness were SPI-1 (*invABCEI*, *sicA*, *sipABD*, and *spaOPQRS*), SPI-2 (*sseC*), SPI-3 (*marT* and *mgtCB*), and SPI-11 (*envF*). Salmonella genes other than SPI essential for fitness under *in vitro* stresses and *in vivo* survival were *atpAEF*, *lepA*, *dam*, *pstB*, *xerC*, *manA*, *phoQ*, *rfaQ*, *rfbBIP*, *rpe*, *trmE*, *rfbIP*, *ompR*, *csgF*, *recG*, *hscA*, *barA*, and putative genes STM14_1138, STM14_3334, STM14_4825, and STM14_5184 (Table 1).

**TABLE 1:** Salmonella genes required for *in vitro* and *in vivo* (enteric and systemic) fitness

Category^*a*^	Genes^*a*^^,^^*b*^	Conditions (*in vitro*, enteric, and systemic)^*c*^^,^^*d*^	COG^*c*^	Protein name
SPI genes				
SPI-1*	*invA*	*Na*, *S*, **C**, **P**, **Ch**, MΦ, SL	U	Needle complex export protein
	*invB*	*Na*, *S*, **C**, **P**, **Ch**, MΦ, SL	U	Secretion chaperone
	*invC*	*Na*, *S*, **C**, **P**, MΦ, PM	NU	ATP synthase SpaL
	*invE*	*Na*, *S*, **C**, **P**, **Ch**, MΦ, SL	U	Invasion protein
	*invI*	*Na*, *S*, **C**, **P**, **Ch**, MΦ, SL	S	Needle complex assembly protein
	*sicA*	*Na*, *S*, **C**, **P**, **Ch**, MΦ, SL	S	Secretion chaperone
	*sipA*	*Na*, *S*, **C**, **P**, **Ch**, MΦ	D	Secreted effector protein
	*sipB*	*Na*, *S*, **C**, **P**, **Ch**, MΦ, SL	U	Translocation machinery component
	*sipD*	*Na*, *S*, **C**, **P**, **Ch**, MΦ	S	Translocation machinery component
	*spaO*	*Na*, *S*, **C**, **P**, **Ch**, MΦ, PM, SL	U	Surface presentation of antigens protein SpaO
	*spaP*	*Na*, *S*, **C**, **P**, **Ch**, MΦ, AM, SL	U	Surface presentation of antigens protein SpaP
	*spaQ*	*Na*, *S*, **C**, **P**, **Ch**, SL	U	Needle complex export protein
	*spaR*	*Na*, *S*, **C**, **P**, **Ch**, MΦ, PM, SL	U	Needle complex export protein
	*spaS*	*Na*, *S*, **C**, **P**, **Ch**, MΦ	U	Surface presentation of antigens protein SpaS
SPI-2*	*sseC*	*pH*, *S*, **C**, **P**, **Ch**, MΦ, SL	S	Translocation machinery component
SPI-3	*marT*	*Na*, **C**, **P**, **Ch**, PM	K	Putative transcriptional regulator
SPI-11	*envF*	*pH*, **C**, **Ch**, MΦ	XX	Putative envelope lipoprotein
Non-SPI genes				
Two-component system	ompR*	*B*, *S*, **C**, **P**, MΦ, SL	T	Osmolality response regulator
	phoQ*	*B*, *S*, *dLB*, **C**, **Ch**, AM, SL	E	Sensor protein PhoQ
	*barA*	*pH*, *S*, **C**, **P**, **Ch**, MΦ	T	Hybrid sensory histidine kinase BarA
O antigen biosynthetic process	*rfbB*	*B*, *S*, **C**, **P**, **Ch**, AM, SL	M	dTDP-glucose-4,6-dehydratase
	*rfbP*	*B*, *S*, *L4*, **C**, **P**, **Ch**, PM	M	Undecaprenol-phosphate galactosephosphotransferase/O-antigen transferase
	*rfbN*	*B*, *S*, **C**, **P**, **Ch**, SL	M	Rhamnosyl transferase
ATP synthase genes*	*atpA*	*PA*, *Na*, *pH*, *H2*, *S*, **C**, **P**, **Ch**, SL	C	F_o_F_1_-ATP synthase subunit alpha
	*atpE*	*PA*, *Na*, *pH*, *H2*, *S*, **P**, **Ch**, MΦ, SL	C	F_o_F_1_-ATP synthase subunit C
	*atpF*	*PA*, *Na*, *pH*, *H2*, *S*, **C**, **Ch**, MΦ	C	F_o_F_1_-ATP synthase subunit B
Mismatch repair	dam*	*pH*, *H2*, *S*, **C**, **P**, **Ch**, AM, SL	L	DNA adenine methylase
Chromosome segregation	xerC*	*PA*, *pH*, *H2*, *S*, **C**, **P**, **Ch**, SL	L	Site-specific tyrosine recombinase XerC
Fructose and mannose metabolism	manA*	*B*, *L4*, *dLB*, **C**, **P**, **Ch**, SL	G	Mannose-6-phosphate isomerase
Carbon metabolism	rpe*	*pH*, *H2*, *S*, **C**, **P**, **Ch**, SL	G	Ribulose-phosphate 3-epimerase
Homologous recombination	*recG*	*B*, *dLB*, **C**, **P**, **Ch**, AM	L	ATP-dependent DNA helicase RecG
ABC transporter	pstB*	*B*, *L4*, *S*, *dLB*, **C**, **P**, **Ch**, SL	P	Phosphate transporter subunit
Translational elongation	lepA*	*B*, *L4*, *S*, *dLB*, **C**, **P**, **Ch**, MΦ, SL	M	GTP-binding protein LepA
Iron-sulfur cluster assembly	*hscA*	*pH*, *H2*, **C**, **Ch**, MΦ, SL	F	Chaperone protein HscA
Others	csgF*	*pH*, **C**, **P**, **Ch**, MΦ, PM	U	Curli assembly protein CsgF
	*rfaQ*	*B*, *L4*, *dLB*, **C**, **P**, **Ch**, SL	M	Lipopolysaccharide core biosynthesis protein
	*rfbI*	*B*, *S*, *dLB*, **C**, **P**, **Ch**, MΦ	GM	CDP-6-deoxy-delta-3,4-glucoseen reductase
	trmE*	*PA*, *H2*, *S*, **C**, **P**, **Ch**, SL	S	tRNA modification GTPase TrmE
Putative protein	STM14_1138	*pH*, **C**, **P**, **Ch**, MΦ	E	Putative transcriptional regulator
	STM14_3334	*pH*, **C**, **P**, SL	S	Putative DNA/RNA helicase
	STM14_4825	*Na*, **C**, **P**, **Ch**, MΦ	H	Coproporphyrinogen III oxidase
	STM_5184	*Na*, **C**, **P**, **Ch**, SL	S	Putative inner membrane protein

a Genes marked with an asterisk (*) have been implicated in vaccine development or drug targeting against a wide range of bacteria.

b The genes listed are required for both *in vitro* and *in vivo* fitness (enteric and systemic infection) (i.e., conditions listed in Fig. 5 and 6).

c COG, cluster of orthologous groups (same as Fig. 2); SPI, Salmonella pathogenicity island; Na, NaCl; H2, H_2_O_2_; S, starvation; C, cattle; P, pig; Ch, chicken; MΦ, macrophage; SL, Sp-Liv; pH, pH 3; B, bile; L4, LB42; AM, A-Mice; PM, P-Mice.

d For conditions, italic font indicates *in vitro*, bold font indicates enteric, and underlining indicates systemic.

Interestingly, most of the common 44 genes required for *in vitro* and *in vivo* (enteric and systemic infection) fitness have been implicated in vaccine or drug target development against a broad spectrum of bacteria; for example, ATP synthase genes (87, 89), *dam* (99, 100), *pstB* (100), *phoQ* (101), *ompR* (102), *xerC* (103), and *rfbBPN* (104), *manA* (105), *rpe* (106), *lepA* (107), *csgF* (108), trmE (109), and SPI-1 and SPI-2 (110) have been used in vaccine development or drug target development (Table 1). Thus, there lies a great potential to explore genes *marBCT*, *envF*, *barA*, *hscA*, *rfaQ*, *rfbI*, and putative proteins STM14_1138, STM14_3334, STM14_4825, and STM_5184 as novel therapeutic and intervention strategies to curb Salmonella infection.

## MATERIALS AND METHODS

### Bacterial strains and growth conditions.

*S.* Typhimurium 14028s, a spontaneous mutant resistant to nalidixic acid (NA), was grown in LB plates or LB medium (BD Difco, Sparks, MD) on a shaking rack at 225 rpm and incubated at 37°C unless otherwise indicated. The single-gene deletion mutants Δ*atpC* and Δ*atpF* in *S*. Typhimurium 14028s strain background were obtained from BEI Resources, NIAID, NIH, Salmonella enterica subsp. *enterica*, strain 14028s (serovar Typhimurium) single-gene deletion mutant library, plate SGD_156/157_Kan, NR-42849. NA (ICN Biomedicals Inc., Aurora OH, USA) and kanamycin (Km; Shelton Scientific Inc. CT, USA) were used at 25 μg/mL and 50 μg/mL, respectively. The *S.* Typhimurium strains were stored in 50% glycerol at −80°C. All procedures involving this bacterial pathogen (biosafety level 2) were conducted according to the protocol approved by the Institutional Biosafety Committee (IBC) at the University of Arkansas.

### Construction of transposon mutant library.

To prepare electrocompetent cells, *S.* Typhimurium 14028s (NA^r^) was grown overnight in 10 mL of LB medium supplemented with NA, which was subsequently diluted 100-fold in 10 mL of 2× yeast extract tryptone (2xYT) medium (BD Difco, Sparks, MD, USA) containing NA and incubated for 3 h on a shaking rack. Bacterial cells were washed 6 times with wash solution (cold 10% glycerol). Centrifugation was done at 8,000 rpm for 1 min at refrigeration temperature (4°C). The bacterial pellet was resuspended gently in 60 μL of wash solution, preventing aeration. One microliter of the EZ-Tn5 <KAN-2> Tnp transposome complex (Epicentre BioTechnologies, Madison, WI, USA) was added to electrocompetent *S.* Typhimurium cells and incubated on ice for 10 min. Then, the mixture was gently transferred to an ice-cold cuvette, avoiding the formation of any air bubbles, and electroporated at 2,450 V. Immediately, 500 μL of super optimal broth with catabolite repression (SOC) was added and incubated for 90 min on a shaking rack at 37°C. The reaction was plated on LB plates supplemented with NA and Km to recover the Tn5 mutants. With 3 electroporations, we were able to collect approximately 350,000 Tn5 mutants and stored them in LB medium with 50% glycerol at −80°C (Fig. 1).

### *In vitro* growth-based selections of transposon mutant library.

*In vitro* selection of the transposon mutant library was done as described previously (111) with some modifications. Briefly, the transposon mutant library was thawed on ice, and an aliquot of 300 μL was added to 60 mL of LB broth with NA and Km (optical density at 600 nm [OD_600_] = 0.131). The library was incubated at 37°C on a shaking rack for 30 min (OD_600_ = 0.135) and centrifuged at 5,500 rpm for 8 min at room temperature. The transposon mutant library pellet was resuspended in 50 mL of 1× PBS (OD_600_ = 0.143), and CFU (4 × 10^7^ CFU/mL) was measured (*t*_1_). This step was included to prepare the mutant cells adapted to LB medium at 37°C, shortening the lag phase in the following selective conditions. Ten-milliliter aliquots were saved from *t*_1_ as an input pool (IP1). The above procedure was repeated to make a technical replicate of IP1 as input pool 2 (IP2). An aliquot of 0.5 mL from *t*_1_ was inoculated in 10 mL of LB, LB containing 100 mM propionate (pH adjusted to pH 7.0; PA), LB with 3% NaCl (NaCl), and LB containing 1 mM H_2_O_2_ (H_2_O_2_). The initial OD_600_ of the inoculated medium was 0.009. We then incubated the libraries on a shaking rack (225 rpm) at 37°C with variable incubation times ranging from 3.75 h to 7 h to reach a mid-logarithmic phase (*t*_2_). The final OD_600_ of all output pools was very similar across the selections and around 0.64 at time point *t*_2_. Input pool and output pool libraries were centrifuged, and the pellets were stored at −80°C for DNA extraction (Fig. 1) (LB, PA, NaCl, and H_2_O_2_).

### *In vitro* survival-based selections of transposon mutant library.

To identify genes negatively selected during starvation, an aliquot of 0.5 mL from *t_1_* was transferred to 10 mL of PBS and incubated at 37°C on a shaking rack for 12 days. On the 12th day, the tube was centrifuged, and the pellet was resuspended in 1 mL of PBS. A 100-μL aliquot was plated and incubated on an LB plate (NA + Km) overnight at 37°C. All colonies were collected in PBS and stored at −80°C for DNA extraction. Whereas for survival at pH 3, 0.5 mL from *t*_1_ was exposed to LB medium adjusted to pH 3 for 30 min at 37°C and immediately transferred to 40 mL of PBS. The cells were centrifuged at 8,000 rpm for 8 min, and the pellet was resuspended in 1 mL of PBS. An aliquot of 250 μL was plated and incubated on an LB plate (NA + Km) overnight at 37°C. All colonies were collected in PBS and stored at −80°C for DNA extraction (Fig. 1) (pH 3 and starvation). Under these two conditions (pH 3 and starvation), a subset of the mutants sensitive to the stressors would lose their cell viability to various degrees, while the mutant cells did not multiply in number. Therefore, to capture only those mutant cells that survived the stressors quantitatively by Tn-seq profiles, the output pools were prepared by recovering the mutant colonies on LB agar plates and combining them in sufficient numbers to represent the populations of all surviving mutants.

### DNA library preparation for Illumina sequencing.

Genomic DNA (gDNA) was extracted from the bacterial cell pellets of input libraries (IP1 and IP2) and output libraries (LB, PA, NaCl, H_2_O_2_, pH 3, and starvation) using a QIAamp DNA minikit (Qiagen, Valencia, CA, USA) following the manufacturer’s protocol and stored at −80°C. Purity and concentration were checked using a Qubit 2.0 fluorometer (Life Technologies, Carlsbad, CA) with Qubit assay kits (double-stranded DNA [dsDNA] broad-range [BR] assay) following the manufacturer’s manual.

The sample for Illumina sequencing was prepared as previously described (15, 17, 18, 112). All DNA primers (Table S1 in the supplemental material) used for Tn-seq library construction were custom designed using Primer3 (v. 0.4.0) (113) and ordered from Integrated DNA Technologies (IDT; Coralville, IA). The simplified diagram for the preparation of the Tn-seq amplicon library is shown in Fig. S1A. Briefly, Tn5 junctions at the right end of the transposon were amplified from gDNA extracted from input and output libraries. The single primer linear extension was done with EZ-Tn5 primer3 using *Taq* DNA polymerase (New England Biolabs, Ipswich, MA, USA). The 50-μL linear PCR extension reaction constituted nuclease-free water (40 μL), ThermoPol buffer (10×, 5 μL), deoxynucleoside triphosphates (dNTPs; 2.5 mM each, 1 μL), EZ-Tn5 primer3 (20 μM, 1 μL), gDNA library (50 ng/μL, 2 μL, ∼100 ng), and *Taq* DNA polymerase (1 μL added during PCR). The PCR cycle consisted of manual hot start with the initial denaturation at 95°C for 2 min and addition of *Taq* DNA polymerase followed by 50 cycles of 95°C for 30 s, 62°C for 45 s, and 72°C for 10 s, which was then followed by a hold at 4°C. The linear PCR products were then purified with a MinElute PCR purification kit (Qiagen, Valencia, CA, USA) and eluted in 10 μL of elution buffer (EB) following the manufacturer’s protocol. Then, deoxycytidine homopolymer tail (C-tail) was added to the 3′ end of the linear PCR extension product using terminal transferase (TdT; New England Biolabs, Ipswich, MA, USA) enzyme as previously described (114, 115). The C-tailing reaction consisted of DNA (linear PCR extension product; 10 μL), TdT buffer (10×, 2 μL), CoCl_2_ (2.5 mM, 2 μL), dCTP (10 mM, 2.4 μL), ddCTP (1 mM, 1 μL), nuclease-free water (1.6 μL), and terminal transferase (1 μL), making a total volume of 20 μL. The reaction mixture was incubated at 37°C for 1 h followed by heat inactivation of the enzyme at 75°C for 20 min on a thermocycler. The C-tailed products were purified using a MinElute PCR purification kit and eluted to 10 μL.

Subsequently, the C-tailed PCR product was enriched with exponential PCR. PCR constituted nuclease-free water (35 μL), ThermoPol buffer (10×, 5 μL), dNTPs (2.5 mM each, 4 μL), IR2 barcoded (BC) primer (with Illumina adapter and barcode; 10 μM, 2 μL), HTM primer (with Illumina adapter; 20 μM, 1 μL), C-tailed DNA (2 μL), and *Taq* DNA polymerase (New England Biolabs; 1 μL), making a total volume of 50 μL. The manual hot start PCR cycle was comprised of 95°C for 2 min, followed by 25 cycles of 95°C for 30 s, 58°C for 45 s, and 72°C for 20 s, trailed by a final extension at 72°C for 10 min.

Finally, the exponential PCR products were heated at 65°C for 15 min and run on 1.5% agarose gels. The Tn-seq library showed a smear pattern, whereas *S.* Typhimurium wild type (negative control) showed almost no amplification (Fig. S1B). The gel was excised ranging from 300 to 500 bp, and DNA was extracted using a QIAquick gel extraction kit (Qiagen, Valencia, CA). The purity and concentration of DNA were measured using a Qubit 2.0 fluorometer. Equal amounts (∼10 ng) of DNA (gel-purified products) from each library were mixed and sent for next-generation sequencing on an Illumina HiSeq 2000 with single-end reads and 100 cycles (Center for Genome Research and Biocomputing, Oregon State University, Corvallis).

### Analysis of Tn-seq data.

Raw reads from HiSeq Illumina sequencing were demultiplexed based on the barcodes to their respective libraries using a custom Perl script. The barcode and transposon sequences were trimmed off from the 5′ end. Consequently, the remaining sequence was Tn5 junction sequences with/without poly(C)-tail. Only 20 bp from the Tn5 junction were kept, discarding most of the poly(C)-tails. The reads were then aligned against the *S.* Typhimurium 14028s complete genome (NC_016856.1) using Bowtie version 0.12.7 (116). The aligned sequence (SAM mapping file) was fed to the ARTIST pipeline to identify conditionally essential genes (CEGs) using Con-ARTIST (27). Briefly, Tn5 insertion frequency was assigned to the *S.* Typhimurium 14028s genome divided into 100-bp window sizes. Uncorrected raw data (nonnormalized) of input and output libraries were normalized and were compared between the matching input and output pool using a Mann-Whitney U test (MWU). The MWU results were used to train the hidden Markov model (HMM) to predict the likelihood of loci that were not required for growth under either condition, essential under both conditions, and depleted in output library (*P* < 0.01). Only the insertions in the central 80% of the gene were considered to eliminate any insertions that may not disrupt the gene functions effectively. The cutoff of a >8-fold decrease was applied as an additional filter for identification of the depleted loci to those genes that showed significant changes (*P* < 0.01).

### Comparative analysis of CEGs between *in vitro* and *in vivo* stressors.

We compared the *in vitro* essential genes identified in this study and our previous study (12) with the previously identified *in vivo* fitness genes. CEGs for acute infection of mice (A-Mice), macrophage survival (MΦ) (9), and persistent infection of mice (P-Mice) (8) were previously identified in *S.* Typhimurium strain SL1344 background. Additionally, Salmonella genes required for gastrointestinal colonization of pigs, calves, and chickens were identified in *S.* Typhimurium strain ST4/74 (10), and those for intraperitoneal infection of mice (Sp-Liv) were reported in *S.* Typhimurium strain 14028s background (11). The CEGs of different strains were searched for the corresponding orthologous genes in *S.* Typhimurium 14028s background using the prokaryotic genome analysis tool (PGAT) (117). To get insight into the phenotypic basis of CEGs required for *in vivo* intestinal colonization of pigs, calves, and chickens, these CEGs were compared with CEGs of *in vitro* host stressors found in the gut (current study: PA, NaCl, and pH 3; reference 12: bile and LB42). Similarly, for the phenotypic basis of CEGs for *in vivo* systemic infection (A-Mice, MΦ, P-Mice, and Sp-Liv), those CEGs were compared to *in vitro* macrophage stressors (current study: H_2_O_2_, NaCl, starvation, and pH 3; reference 12: dLB). Only the CEGs that were common between at least one of the *in vitro* host stressors and at least one of *in vivo* infections were identified and included in the comparative analysis.

### Mutant phenotypic testing.

For growth assays, overnight cultures of bacteria were prepared as described above and inoculated in LB medium, LB medium containing 100 mM propionate (pH 7), LB medium containing 3% NaCl, and LB medium containing 1 mM H_2_O_2_. The OD_600_ was monitored every 1 h using a Tecan Infinite M200 plate reader (Tecan Trading AG, Switzerland) during incubation at 37°C with shaking (200 rpm) for 16 h. For survival assays, 0.1 mL of the overnight culture was washed 3 times in PBS (pH 7.0) and inoculated in 10 mL of 1× PBS (pH 7.0) and glycine-HCl buffer (pH 3.0) for starvation survival and pH 3.0 survival, respectively, and the cell suspensions were incubated at 37°C. For the starvation assay, an aliquot of the sample was collected at days 0, 4, and 7, and viable cells were enumerated by plating 10-fold serial dilutions on LB agar plates. For the pH 3 survival assay, an aliquot of the sample was collected at 0, 1.5, and 2 h, and viable cells were enumerated by plating 10-fold serial dilutions on LB agar plates. All of the assays were performed in at least 3 replications.

### Data availability.

Sequencing data for Tn-seq analysis in this study are available on the NCBI Sequence Read Archive under BioProject number PRJNA385835.
