# Supplementary material for: Genetic Determinants in Salmonella enterica Serotype Typhimurium Required for Overcoming In Vitro Stressors in the Mimicking Host Environment
Source: Microbiol Spectr. 2021 Dec 8;9(3):e00155-21. doi: 10.1128/Spectrum.00155-21 (PMC8653844; doi:10.1128/Spectrum.00155-21)
Supplement: SUPPLEMENTAL FILE 2 — Supplemental material. Download Spectrum.00155-21-s0002.pdf, PDF file, 1.7 MB [file spectrum.00155-21-s0002.pdf]

Supplemental Materials

Genetic determinants in *Salmonella enterica* serotype Typhimurium required for overcoming *in vitro* stressors in the mimicking host environment

Rabindra K. Mandal[1*], Tieshan Jiang[1] and Young Min Kwon[1, 2]

[1]Department of Poultry Science, University of Arkansas, Fayetteville, AR, 72701

[2]Cell and Molecular Biology Program, University of Arkansas, Fayetteville, AR, 72701

*Corresponding author's current address:

Ryan White Center for Pediatric Infectious Diseases and Global Health

Department of Pediatrics

Indiana University School of Medicine

1044 West Walnut Street | R4 451A

Indianapolis, IN 46202

Phone: (479) 276-2203

rmandal@iu.edu

Figure S1. Preparation of Tn-seq amplicon library for Illumina sequencing. **(A)** Genomic DNA of Tn5 mutant library was linearly extended using Tn-specific primer1 (Ez-Tn5 primer3 in **Table S1**). Then C-tail was attached to the 3' end of purified single-stranded DNA. The C-tailed product was purified and exponential PCR was performed using Tn-specific primer2 (Barcoded primers, IR2-IS-B7~B15, in **Table S1**) and C-tail specific primer (HTM-Primer in **Table S1**) with the attached Illumina adapters). **(B)** Exponentially amplified DNA was then run on 1.5% agarose gel. DNA from 300bp to 500bp was extracted from the gel and sent for Illumina HiSeq sequencing. [M: Hi-Lo DNA marker; 1, 2, 3, 4: Tn5 mutant libraries; and C: negative control (*S.* Typhimurium 14028s wild type)].

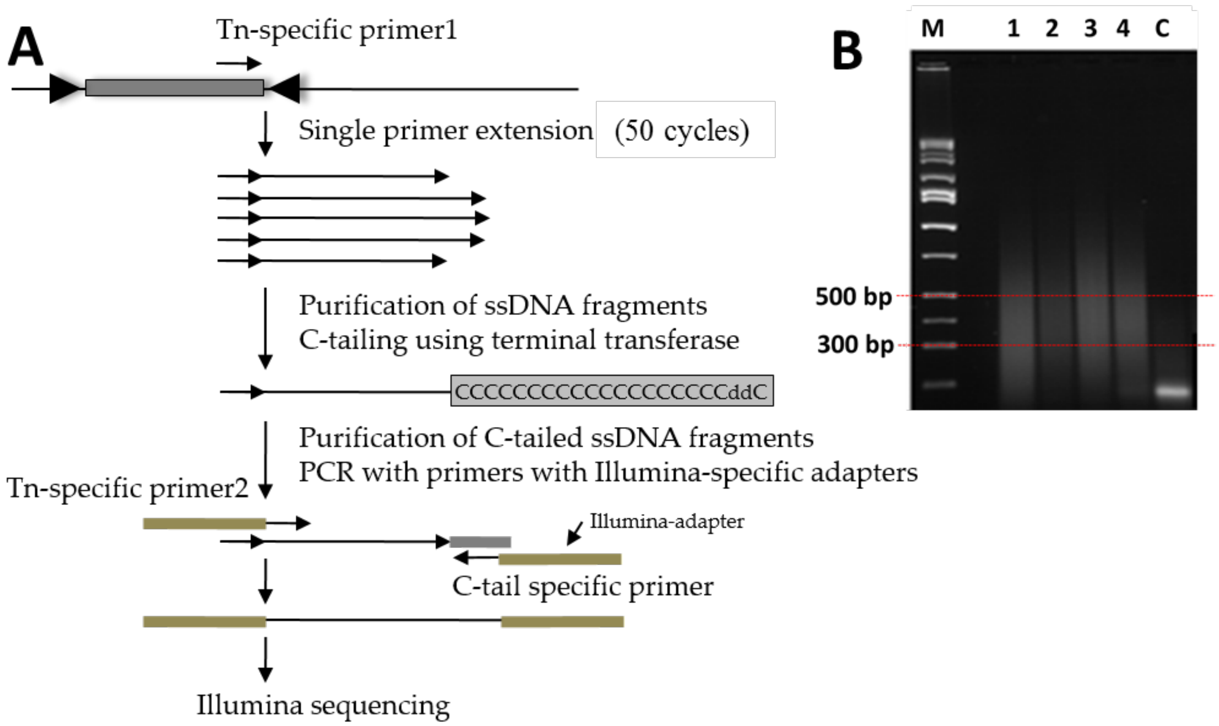

Figure S2. Growth curves of *S.* Typhimurium 14028s wild type in LB medium (Control), LB-propionate, LB-NaCl, and LB- H_2O_2. LB-propionate, LB-NaCl, and LB- H_2O_2 represent LB media supplemented with 100 mM propionate, 3% NaCl, and 1 mM H_2O_2, respectively. OD_{600} was monitored every 10 min using Tecan Infinite M200 plate reader (Tecan Trading AG, Switzerland) during incubation at 37°C with shaking (200 rpm) for 24 hrs.

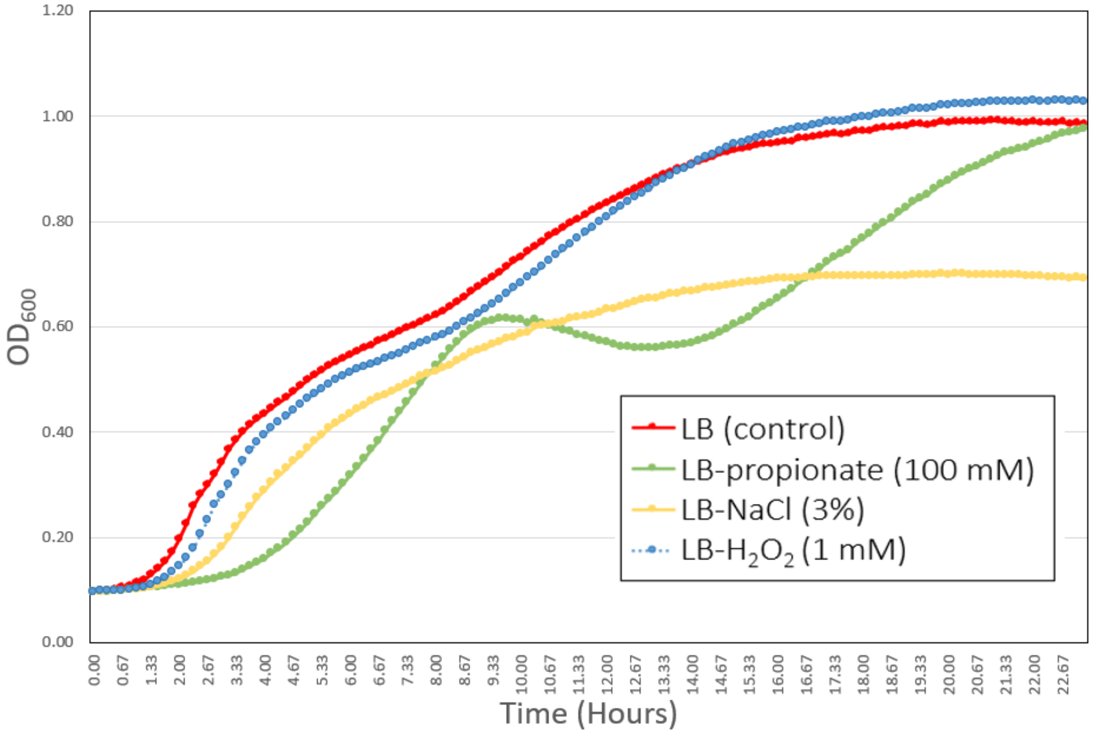

Figure S3. Summary of the Illumina sequencing reads and comparison among the Tn5 mutant libraries. **(A)** The bar graph shows the number of the Illumina sequencing reads in each Tn5 libraries: after barcode sorting (blue color), unique insertions (orange), mean (grey) and median reads (yellow) for each unique insertion. **(B)** The scatter plots display the Spearman correlation (R^2) between a pair of the indicated Tn5 mutant libraries based on read count per 100bp window across the genome ($p < 0.0001$).

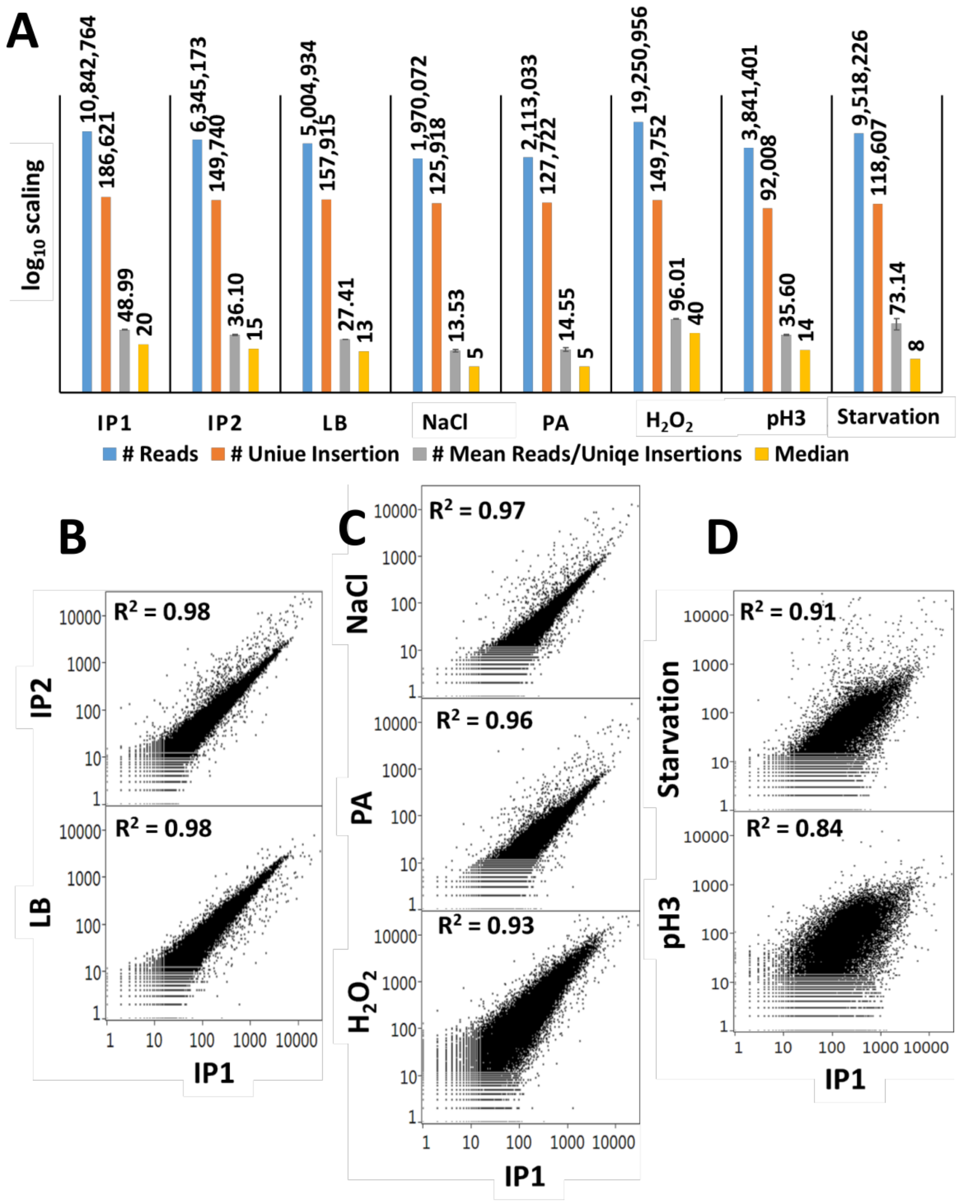

Figure S4. Overlay plot displays global view of genome-wide quantitative distribution of Tn5 insertion read count for all samples. X-axis: the genomic coordinates and Y-axis: Number of read count per 100 bp scaled in $\log_{10}$.

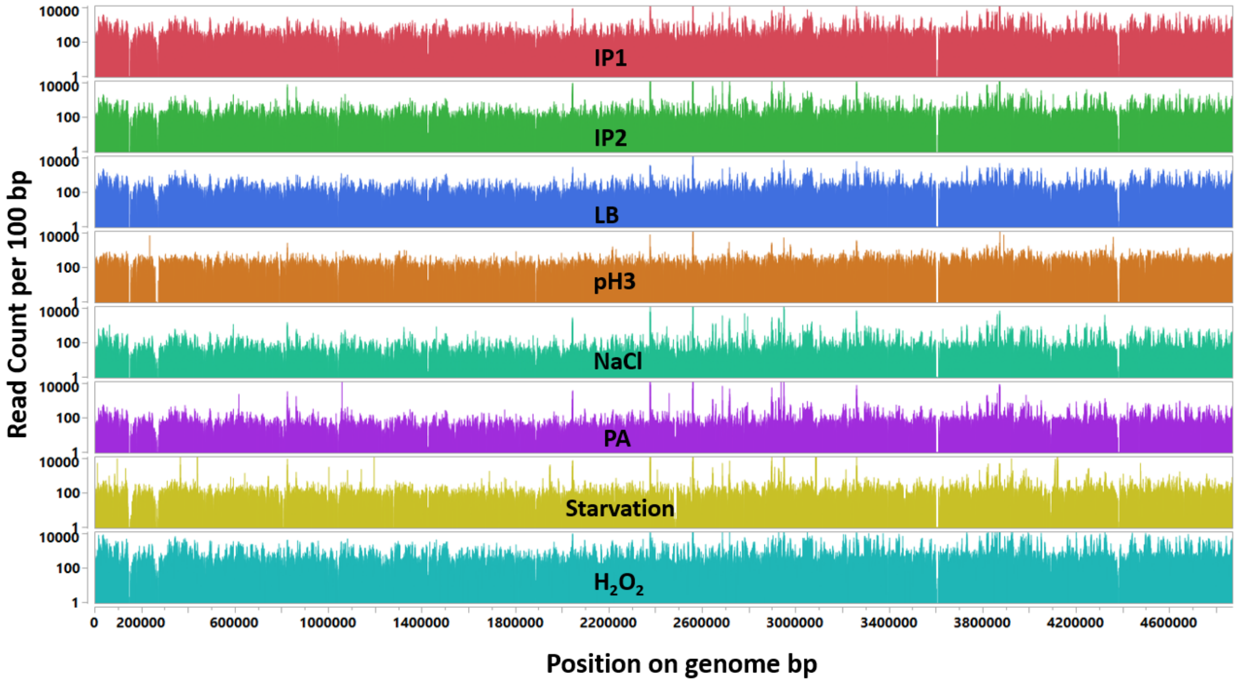

Figure S5. The Tn-seq profiles around the selected genomic regions. **(A)** *Salmonella* pathogenicity island 1 (SPI-1) genes encoding type III secretion system (TTSS). Screen shot image produced using Integrative Genomics Viewer (IGV) showing raw read coverage [100-600] in the 7 conditions. (Blue asterisk: conditionally essential in NaCl and Starvation; and Red asterisk: conditionally essential in Starvation only). **(B)** CpxAR were conditionally essential in Starvation only.

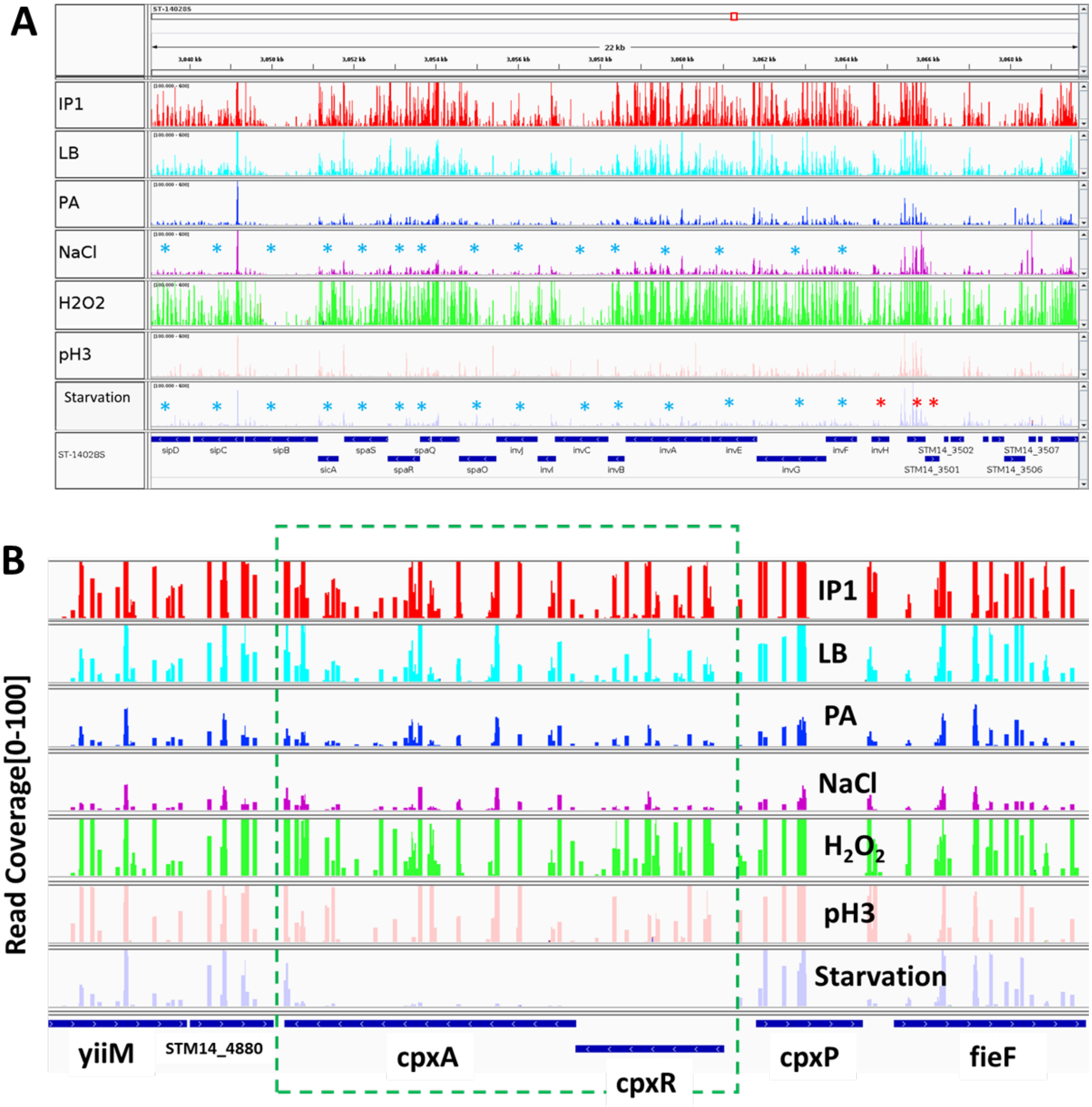

Figure S6. Comparison of the overlapping set of the conditionally essential genes of *S*. Typhimurium 14028s (this study) with the essential genes of *S*. Typhimurium SL3261 and *S*. Typhimurium LT2 identified in previous studies.

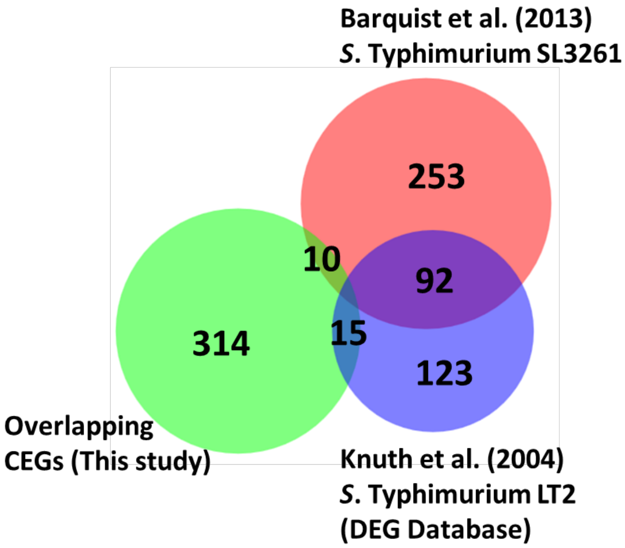

Figure S7. Venn diagram showing the total of 339 conditionally essential genes (CEGs) in *S.* Typhimurium 14028s identified in this study that are required for fitness in at least one of the five stress conditions (PA, NaCl, H_2O_2, Starvation, and pH3)

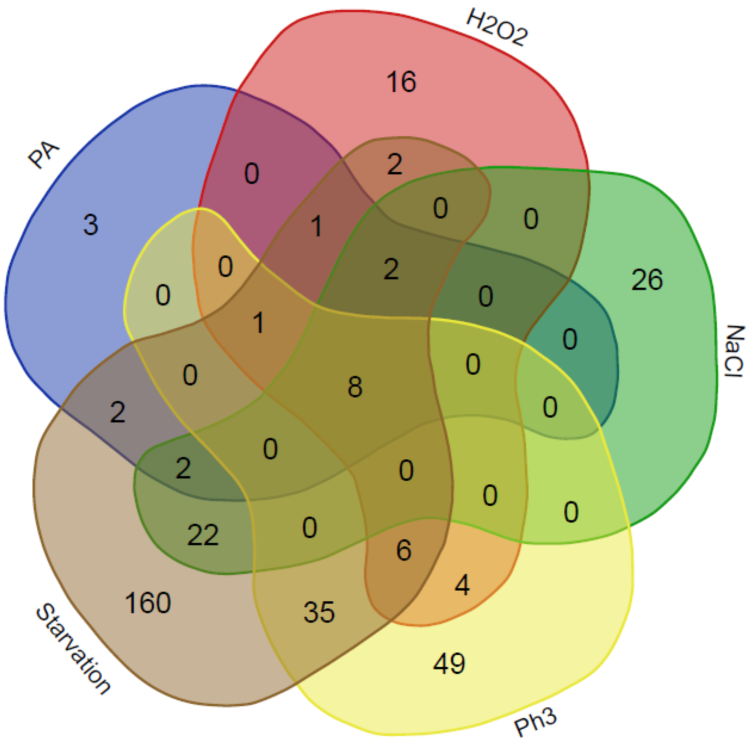

Figure S8. Genes required for enteric infection, systemic infection and *in vitro* fitness. Venn diagram shows the number of the shared genes between *in vitro* vs *in vivo* (Enteric) (135 CEGs shown in **Figure 4**) and *in vitro* vs *in vivo* (Systemic) (130 CEGs shown **Figure 5**). The list of 44 genes required for all *in vitro* fitness, enteric infection and systemic infection are shown in **Table 1**.

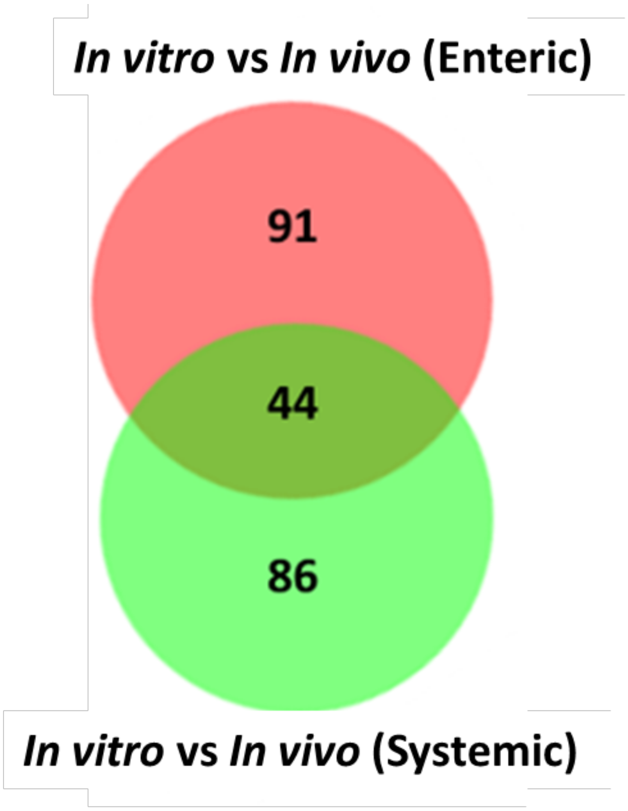

List of Supplementary Tables

Table S1. Oligonucleotides used in this study.

Table S2. All conditionally essential genes (CEGs) in *S.* Typhimurium 14028s identified in this study.

Table S3. Comparison of the conditionally essential genes (CEGs) in *S.* Typhimurium 14028s identified in this study across the 5 stress conditions.

Table S4. The conditionally essential genes (CEGs) in *S.* Typhimurium 14028s identified in this study that are located in Salmonella Pathogenicity Islands.

Table S5. Comparison of the conditionally essential genes (CEGs) of *S.* Typhimurium 14028s identified in this study with the essential genes of *S*. Typhimurium identified from previous studies

Table S6. The conditionally essential genes (CEGs) required for growth (PA, NaCl, Bile and LB42) or survival (pH3) in the presence of the *in vitro* host stressors that are also required for enteric infection in farm animals (cattle, pig, and chicken)

Table S7. The conditionally essential genes (CEGs) required for growth (H_2O_2, NaCl, and dLB) or survival (pH3 and starvation) in the presence of the *in vitro* host stressors that are also required for systemic infection (MΦ, A-Mice, P-Mice, and Sp-Liv).
